# Supplementary material for: Being noisy in a crowd: Differential selective pressure on gene expression noise in model gene regulatory networks
Source: PLoS Comput Biol. 2023 Apr 20;19(4):e1010982. doi: 10.1371/journal.pcbi.1010982 (PMC10118199; doi:10.1371/journal.pcbi.1010982)
Supplement: S1 Text — (PDF) [file pcbi.1010982.s001.pdf]

Supplementary Information:

Being noisy in a crowd: differential selective pressure on gene expression noise in model gene regulatory networks

Nataša Puzović[1*], Tanvi Madaan [1], Julien Dutheil[1,2]

1 Molecular Systems Evolution Research Group, Max Planck Institute for Evolutionary Biology, Plön, Schleswig-Holstein, Germany
2 Institut des sciences de l'évolution, Montpellier, Languedoc-Roussillon, France

* puzovic@evolbio.mpg.de

This document contains all supplementary figures and information referenced in the main text. The simulation results data and the code necessary to reproduce all figures is available at `https://doi.org/10.5281/zenodo.6939845`, together with the the code necessary to generate all raw simulation files.

1 Gene regulatory network model and evolutionary model

1.1 Parameters

The parameter values used for the gene regulatory network model and evolutionary simulations and their descriptions are shown in Table S1.

Table S1. Parameters used in the simulations.

Parameter	Symbol	Value	Description
Number of nodes in the network	n	40	Number of genes in the gene regulatory network
Network density	d	0.05	Proportion of potential connections in the network
Regulatory matrix	$W = (w_{ij})_{1\leq i\leq n,\, 1\leq j\leq n}$	see Supp. Data	Regulatory relationships in the gene regulatory network
Intrinsic noise	$\{\eta_i^{\text{int}}\}_{1\leq i\leq n}$	100	Gene-specific noise of each gene
Basal expression levels	$\{S_i^{basal}\}_{1\leq i\leq n}$	$\{20, ..., 20\}$	Constitutive expression level
Number of timesteps for genotype realization	T_r	50	Number of timesteps the expression levels are updated
Minimal expression level	s_{min}	0	Minimal expression level
Maximal expression level	s_{max}	100	Minimal expression level
Number of timesteps to check oscillatory dynamics	τ	10	Time window to apply oscillation criterion
Maximal allowed fluctuation in gene expression levels	ϵ	$1e^{-06}$	Criterion to check oscillatory dynamics
Population size	N	1,000	Number of individuals in a population
Number of generations	T	10,000	Length of the evolutionary simulation in generations
Optimal expression levels (for network establishment)	$\{s_i^{opt}\}_{1\leq i\leq n}$	$\{50, ..., 50\}$	Expression levels that correspond to maximum fitness
Mutation rate (regulatory interactions)	μ_w	0.05	Mutation probability of a regulatory interaction, per interaction, per repl. event
Mutation value mean (regulatory interactions)	m_w	0	Mean of normal distribution from which mutation values are drawn
Mutation value variance (regulatory interactions)	v_w	2	Variance of normal distribution from which mutation values are drawn
Selective pressures	$\{\rho_i\}_{1\leq i\leq n}$	$\{1, ..., 1\}$	Contribution of expression level to fitness
Mutation rate (intrinsic noise)	μ_η	0.01	Mutation probability of intrinsic noise, per gene, per repl. event
Mutation value mean (intrinsic noise)	m_η	100	Mean of normal distribution from which mutation values are drawn
Mutation value variance (intrinsic noise)	v_η	40	Variance of normal distribution from which mutation values are drawn
Recombination rate	r	0.05	Probability of offspring entering the recombination process

1.2 Robustness of network realization

In the simulations performed in this study the gene regulatory network model was realized into the phenotype by synchronously updating the expression levels of all genes in every time step. To test whether the time of updating changes the steady state expression levels, we compared steady state expression levels of 200 samples of 20-gene random networks realized without noise synchronously and asynchronously. Synchronous updating was performed by updating the expression level of all genes in the network at every time step for T_r time steps ($T_r = 50$). Asynchronous updating was performed by randomly choosing a gene in every time step and updating only its expression levels, for $n \times T_r = 1000$ time steps. We realized each network topology 1000 times synchronously and asynchronously and measured the Hamming distance between the expression level vectors in the last time step. Two expression level values were deemed identical if their difference was less than 0.001. In 92% of cases the synchronous and asynchronous realizations of the same network configuration had a Hamming distance of 0 (Fig S1A). The mean expression level, expression variance, CV, noise and Fano factor were highly correlated between the synchronous and asynchronous realizations (Fig S1B-F). Examples of expression level dynamics of deterministic and stochastic realizations with synchronous and asynchronous updating schemes in one network are shown in Fig S2.

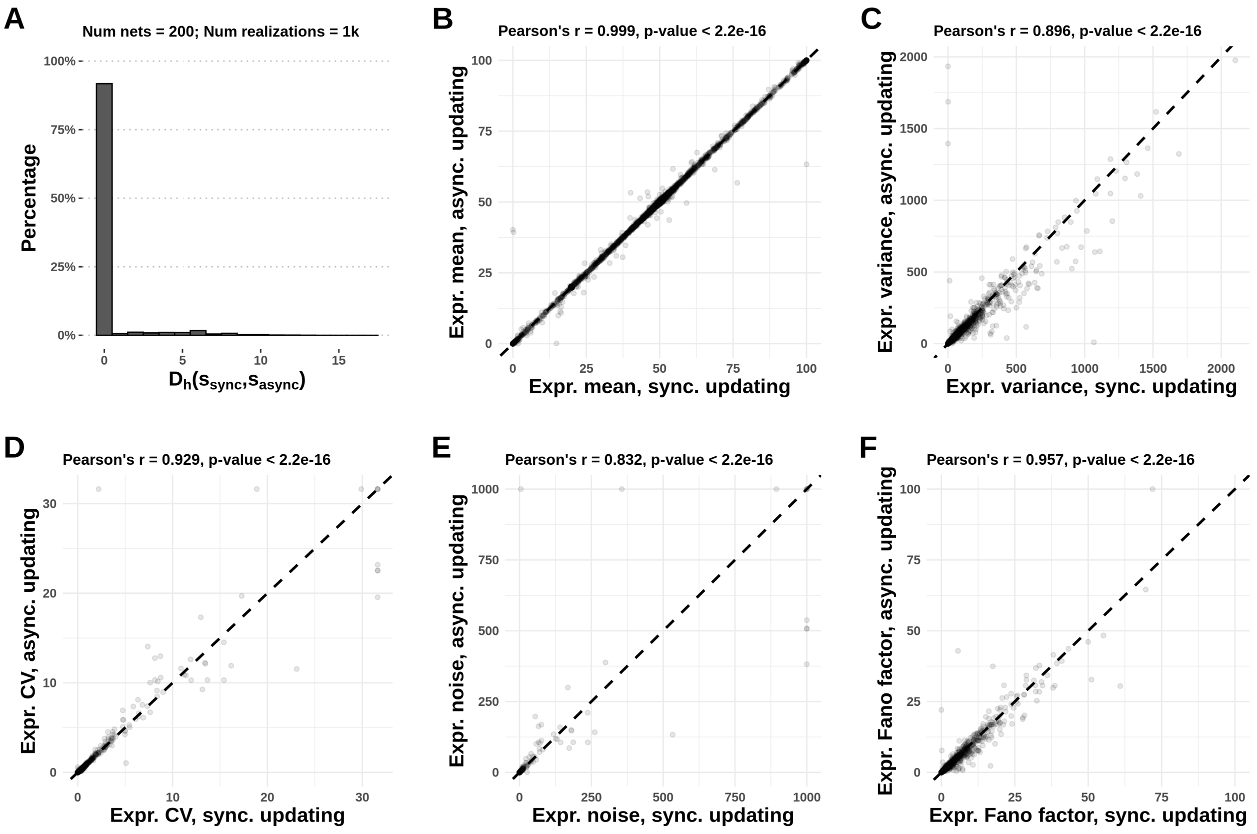

Fig S1. Network realization is robust to synchronous or asynchronous expression level updating mode during network realization. A - Hamming distance between the steady states of synchronously and asynchronously realized networks. Expression level values between synchronous and asynchronous realizations were deemed identical if their difference was less than 0.001. **B** - Mean expression level of populations of synchronously and asynchronously realized networks. **C-F** - Expression level variance, CV, noise and Fano factor of genes from populations of synchronously and asynchronously realized networks.

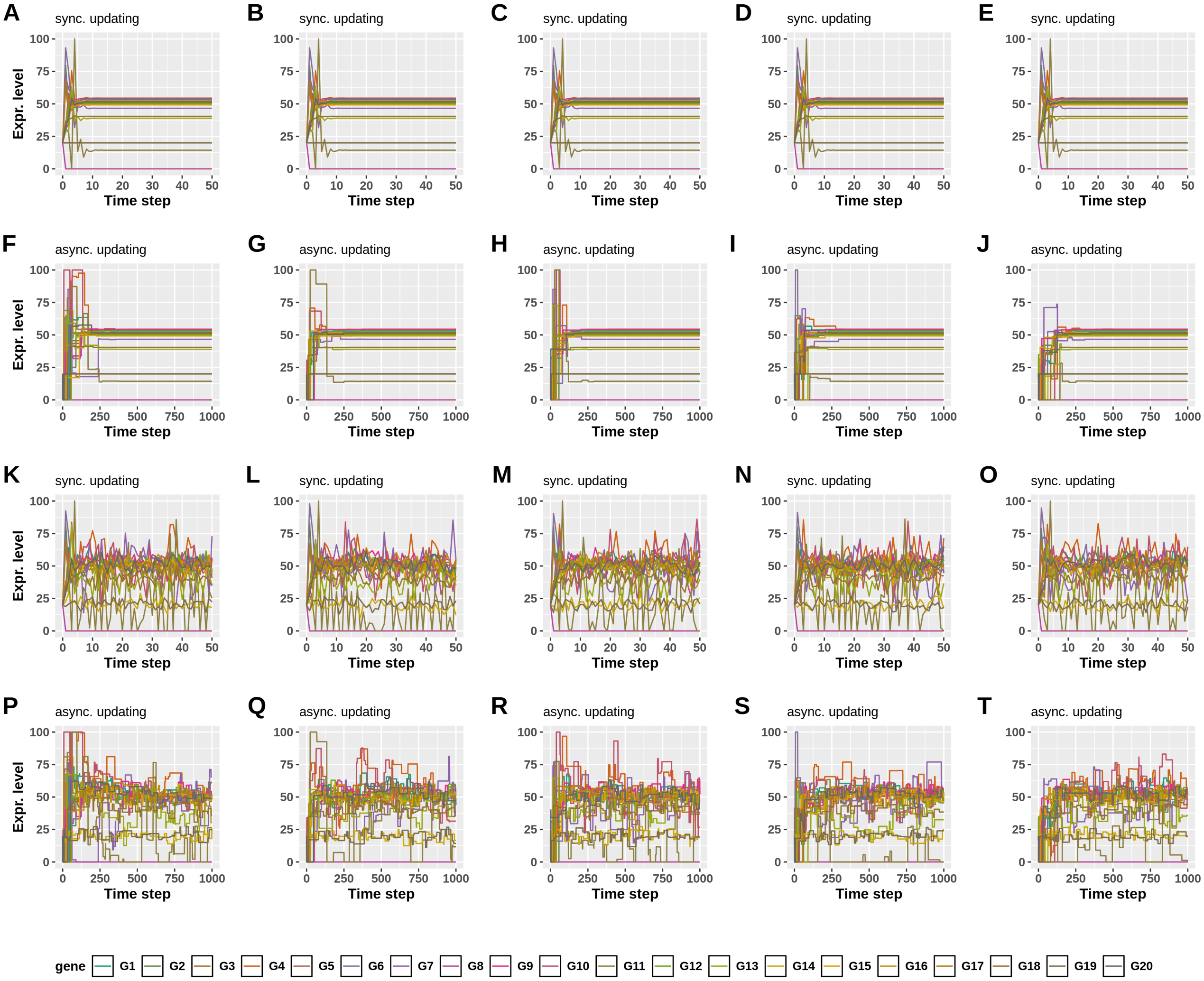

Fig S2. Examples of expression level dynamics in realizations of the same network with different expression level updating modes and noise levels. A-E Five non-noisy realizations with synchronous expression level updating. Since there is no random component in the realization, there are no differences between the realizations. **F-J** Five non-noisy realizations with asynchronous expression level updating. The expression level of a randomly chosen gene is updated in each timestep. Consequently, even though there is no intrinsic expression noise, the dynamics differ between the five realizations, but they reach the same steady state as in the synchronously updated realizations. **K-O** Five noisy realizations with synchronous expression level updating. The mean of the gene expression levels equals the steady state expression levels of the realizations without noise. **P-T** Five noisy realizations with asynchronous expression level updating.

1.3 Convergence of expression levels during network establishment

An optimal expression level of $s_{opt} = 50$ is imposed on all genes during network establishment to find network configurations which have intermediate expression levels. At the end of the network establishment process, 68% (54333/80000) of genes in the fittest networks had a steady state expression level in the range of $s_{opt} \pm \frac{s_{opt}}{2}$ (Fig. S3).

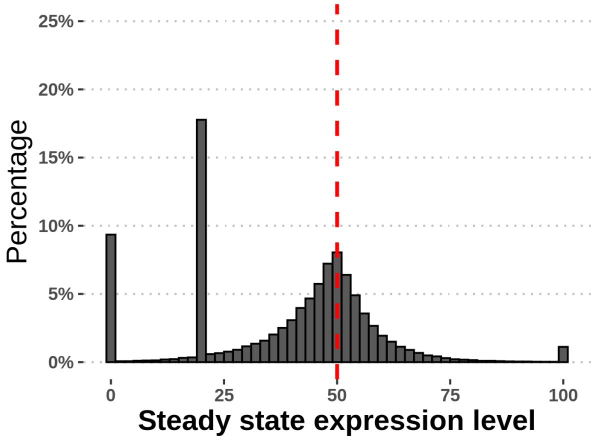

Fig S3. Most genes have intermediate steady state expression level after the network establishment process. Histogram of steady state expression levels after the network establishment process. Dataset consists of 80,000 genes from 2000 random 40-gene network topologies. The peak at $s = 20$ indicates genes which are not activated by other genes and are expressed only at the basal level of $\{S_i^{basal}\}_{1 \leq i \leq n} = \{20, ..., 20\}$. Red dashed line indicates the optimal expression level.

1.4 Population size

We tested the effect of the population size on selective pressure by simulating the evolution of a dataset of 500 network topologies with different population sizes. We find that increasing the population size increases the selective pressure acting on constituent genes (Fig S4). A population size of 1000 was chosen for the main simulations in this study.

1.5 Stability of mean expression level

We imposed stabilizing selection on gene expression levels and observed a repeatable pattern of reduction of gene expression variance. The mean expression level was stable (Fig S5A) throughout evolution, meaning that the adapted populations had a higher fitness due to a reduction of gene expression variance, not changes in expression mean.

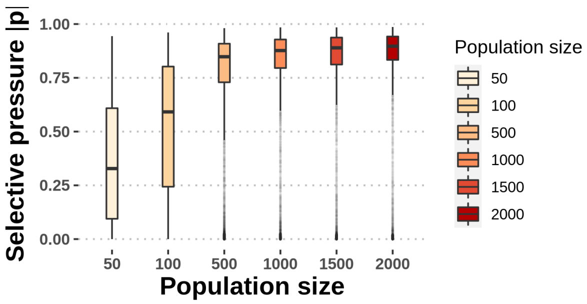

Fig S4. Increasing the population size increases the selective pressure on genes under stabilizing selection on gene expression level. Dataset consists of 20,000 genes from 500 random 40-gene network topologies.

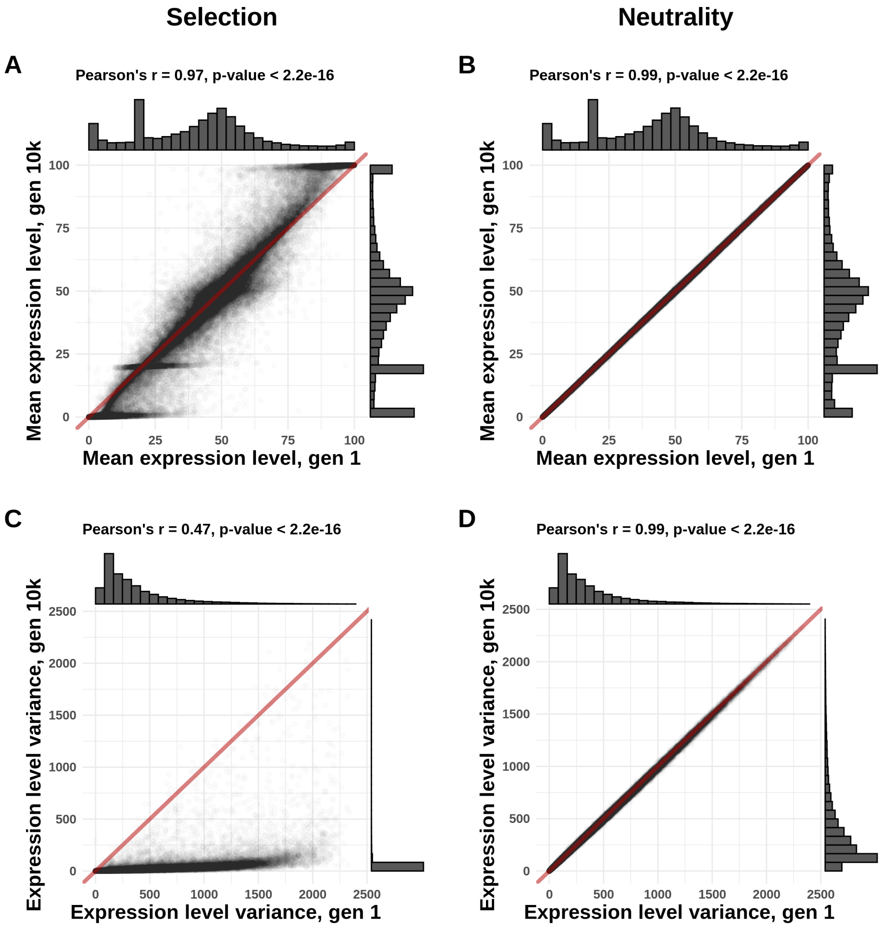

Fig S5. Mean expression level does not change after noise evolution under stabilizing selection on gene expression levels. A, B - Mean expression level in the first and last generation of populations evolved under selection (A) and neutrality (B). **C, D** - Expression variance in the first and last generation of populations evolved under selection (C) and neutrality (D). Red lines indicate lines with a slope of 1.
