# Supplementary material for: Being noisy in a crowd: Differential selective pressure on gene expression noise in model gene regulatory networks
Source: PLoS Comput Biol. 2023 Apr 20;19(4):e1010982. doi: 10.1371/journal.pcbi.1010982 (PMC10118199; doi:10.1371/journal.pcbi.1010982)
Supplement: S2 Text — (PDF) [file pcbi.1010982.s002.pdf]

2 Network centrality metrics

To measure the centrality of nodes in the gene networks, we computed 19 node-level centrality measures. These centrality measures are: degree, indegree, outdegree, closeness, betweenness, eigenvector centrality, node strength, instrength, outstrength, hub score, authority including weights, authority excluding weights, absolute node strength, absolute instrength, and absolute outstrength, flow betweenness, load centrality, information centrality, and stress centrality. These measures were heavily intercorrelated and correlated with the expression noise metrics - expression noise, change of expression noise after selection, and selective pressure (Fig S6). We also computed 12 graph-level centrality measures to study the effects of the global topology on the average selective pressure. These measures are: diameter, mean path distance, degree assortativity, degree centralization, indegree centralization, outdegree centalization, closeness centralization, betweenness centralization, average degree, average indegree, and average outdegree. The global network metrics were intercorrelated, as well (Fig S7). In the study of the effects of network centrality on evolvability of gene-specific expression noise we focused on instrength and outstrength as node-level centrality measures, and summarized the 12 graph-level measures into two synthetic independent variables using principal component analysis.

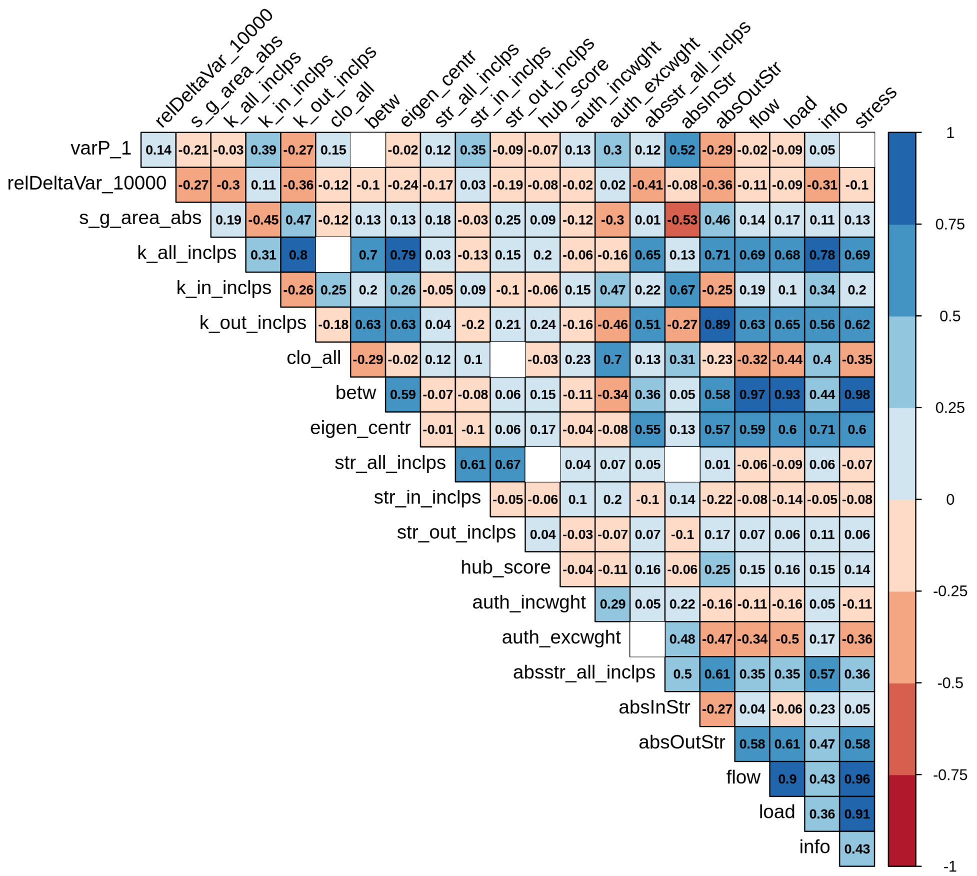

Fig S6. Correlation matrix of node-level network centrality metrics and expression noise metrics. Spearman's rank correlation coefficients shown in the cells. Empty cells indicate a non-significant p-value (p-value > 0.05). Abbreviations: varP_1 - expression variance in the first generation; relDeltaVar_10000 - relative change of expression variance between the first and generation 10,000; s_g_area_abs - selective pressure on each node; k_all_inclps - degree; k_in_inclps - indegree; k_out_inclps - outdegree; clo_all - closeness; betw - betweenness; eigen_centr - eigenvector centrality; str_all_inclps - node strength; str_in_inclps - instrength; str_out_inclps - outstrength; hub_score - hub score; auth_incwght - authority including weights; auth_excwght - authority excluding weights; absstr_all_inclps - absolute strength; absInStr - absolute instrength; absOutStr - absolute outstrength; flow - flow betweenness; load - load centrality; info - information centrality; stress - stress centrality. Dataset consists of 148,886 genes from 2,000 random network topologes.

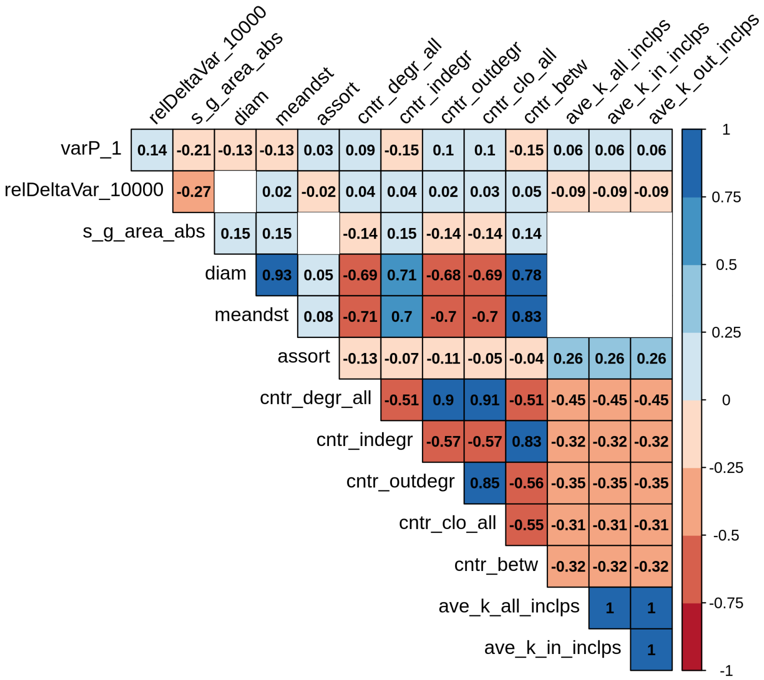

Fig S7. Correlation matrix of graph-level network centrality metrics and expression noise metrics. Spearman's rank correlation coefficients shown in the cells. Empty cells indicate a non-significant p-value (p-value > 0.05). Abbreviations: varP_1 - expression variance in the first generation; relDeltaVar_10000 - relative change of expression variance between the first and generation 10,000; s_g_area_abs - selective pressure on each node; diam - diameter; meandst - mean path distance; assort - degree assortativity; cntr_degr_all - degree centralization; cntr_indegr - indegree centralization; cntr_outdegr - outdegree centralization; cntr_clo_all - closeness centralization; cntr_betw - betweenness centralization; ave_k_all_inclps - average degree; ave_k_in_inclps - average indegree; ave_k_out_inclps - average outdegree. Dataset consists of 148,886 genes from 2,000 random network topologes..

2.1 Colinearity between instrength and outstrength

The predictor variables used in statistical modelling in the main results, node instrength and outstrength, were correlated (Spearman's ρ = -0.17, p-value $< 2.2 \times 10^{-16}$, Fig S8A-B). This correlation is due to the distributions of in and out nodes being non independent: the more in-connections has, the less out-connections. We also observed that a part of the residuals non-normality may be due to points with a value of zero in one of the two predictor variables. As a control, we rerun the entire analysis on two additional filtered datasets. In the first one, we kept only genes with zero values of either instrength or outstrength, *i.e.* this dataset consisted of only regulators and target genes. Instrength and outstrength were more correlated in the first filtered dataset (Spearman's ρ = -0.86, p-value $< 2.2 \times 10^{-16}$, Fig S8C) than in the unfiltered dataset. In the second filtered dataset, we removed all genes that had a zero value of either instrength or outstrength, *i.e.* this dataset consisted of genes that are both regulators and regulated. Instrength and outstrength were less correlated in the second filtered dataset (Spearman's ρ = -0.03, p-value $< 2.2 \times 10^{-16}$, Fig S8C) than in the unfiltered dataset, and this filtering somewhat reduced the heteroskedasticity of the Pearson's residuals in the statistical models. The same pattern of effects and significance of instrength and outstrength was observed in the filtered datasets as in the main dataset, indicating that our conclusions are robust to the heteroskedasticity of Pearson's residuals and collinearity between the explanatory variables. The results of all statistical models are summarized in Table S7 in Section 5.

2.2 PCA of global network metrics

To investigate the effects of the intercorrelated graph-level network centrality metrics on noise propagation and noise evolution, we performed a principal component analysis (PCA) to construct independent summary variables representing graph-level network centrality metrics. The first two dimensions of the PCA expressed 85.4% of the total data inertia (Fig S9A), so we chose the first two principal components (PCs) as synthetic explanatory variables in linear mixed-effects models in the main results. The loadings of the first two PCs are shown in Fig S9B. The loading of the first synthetic variable (PC1) is dominated by negative loadings of diameter and mean path distance, and the centralization measures, namely positive loadings of outdegree and closeness centralization and negative loadings of indegree and betweenness centralization. The loading of the second synthetic variable (PC2) is dominated by the negative loading of the average degree, average indegree and average outdegree measures. For easier interpretation, the sign of the PCs has been switched in the statistical modelling shown in the main text.

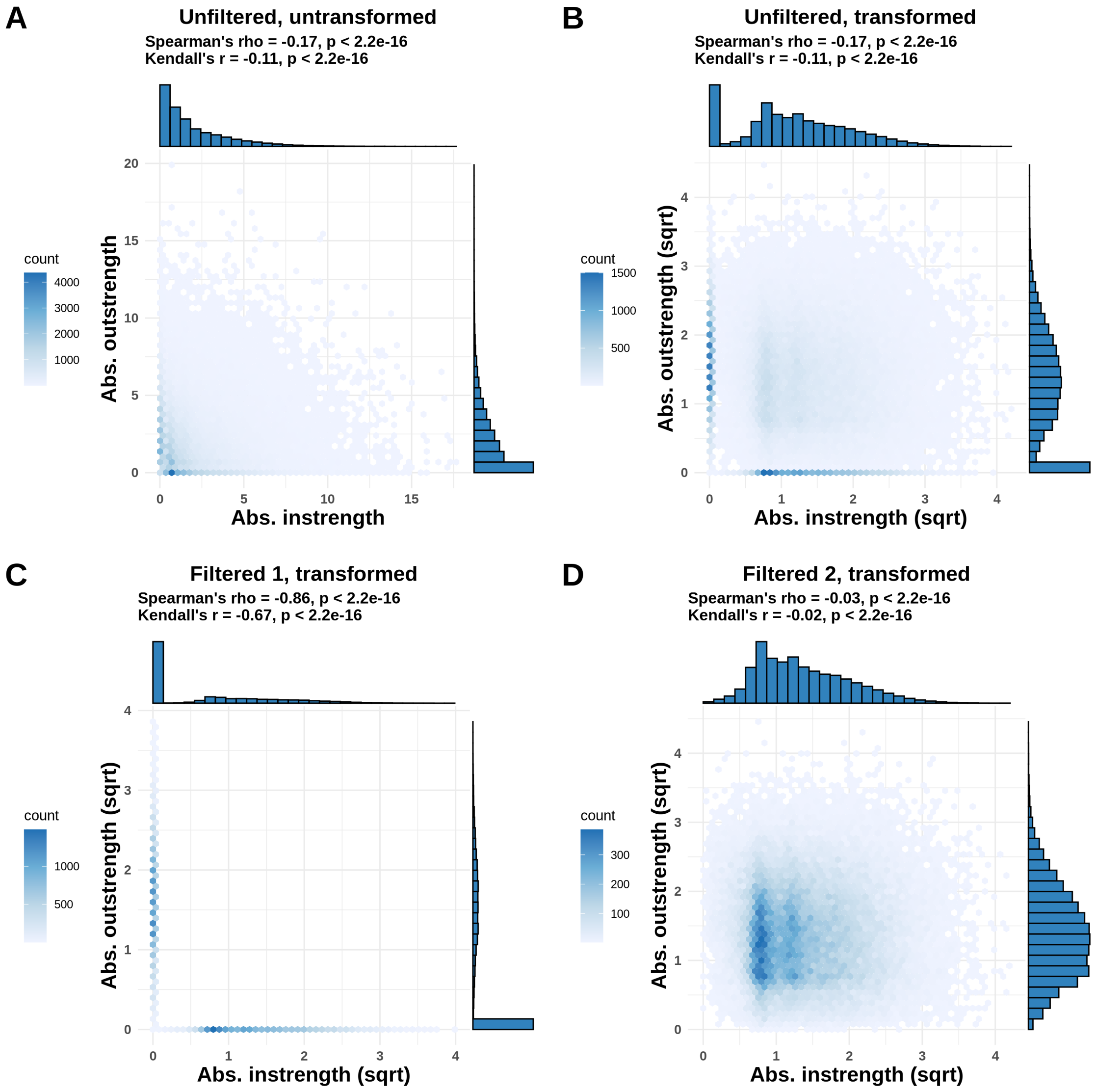

Fig S8. Correlations between node instrength and outstrength in unfiltered and filtered datasets. **A** - Correlation between instrength and outstrength in the unfiltered dataset. Dataset consists of 148,886 genes from 2,000 random network topologies. **B** - Correlation between square-root transformed instrength and outstrength in the unfiltered dataset. **C** - Correlation between square-root transformed instrength and outstrength in the filtered dataset. Dataset consists of 43,214 genes from 2,000 random network topologies. **D** - Correlation between square-root transformed instrength and outstrength in the filtered dataset. The dataset consists of 105,672 genes from 2,000 random network topologies.

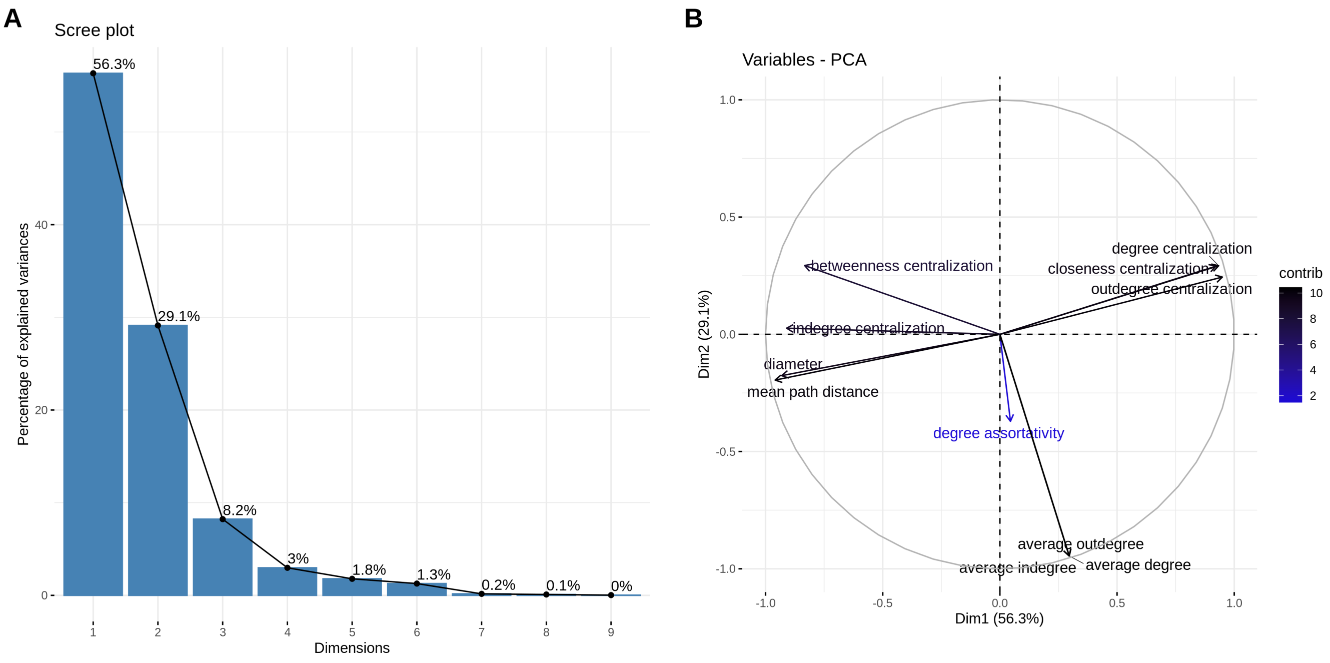

Fig S9. Principal component analysis of the graph-level network centrality metrics. A - Scree plot depicting the percentage of total variance explained by each principal component. The first two principal components express 85.4% of the total inertia. **B** - Correlation circle showing the loadings of the first two principal components.
