# Supplementary material for: Being noisy in a crowd: Differential selective pressure on gene expression noise in model gene regulatory networks
Source: PLoS Comput Biol. 2023 Apr 20;19(4):e1010982. doi: 10.1371/journal.pcbi.1010982 (PMC10118199; doi:10.1371/journal.pcbi.1010982)
Supplement: S3 Text — (PDF) [file pcbi.1010982.s003.pdf]

3 Diagnostics of statistical models

3.1 GLMM: Noise propagation

To investigate whether noise propagation is captured by our gene regulatory network model, we fitted a linear mixed-effects model with the following formula:

$$y = X\beta + Zu + \epsilon, \quad (1)$$

where the outcome variable, y, is a column vector of the expression variance of each node; X is a matrix of two explanatory fixed-effects variables, node instrength and node outstrength, β is a column vector of the two fixed-effects coefficients; Z is the column vector for the design of the random effect variable, network topology sample, and the number of groups equivalent to the number of network topology samples; u is a column vector with the random-effects coefficient for each group (network topology sample); ϵ is a column vector with the residuals. When fitting a model with the assumption of constant variance, the Pearson's residuals were heteroskedastic (Fig S10A). We fitted models with different variance structures and based on Akaike's Information Criterion chose the model with the exponential function of the node instrength as the variance structure. Pearson's residuals of the chosen and all other fitted models are shown in Fig S10B-E. Changing the variance structure did not change the significance or the effect of the fixed variables (Table S2). The variance inflation factor (VIF), a measure of collinearity of explanatory variables, was 1.08. A VIF value lower than 3 indicates that the statistical significance of the inferred effects is reliable in spite of collinearity (1).

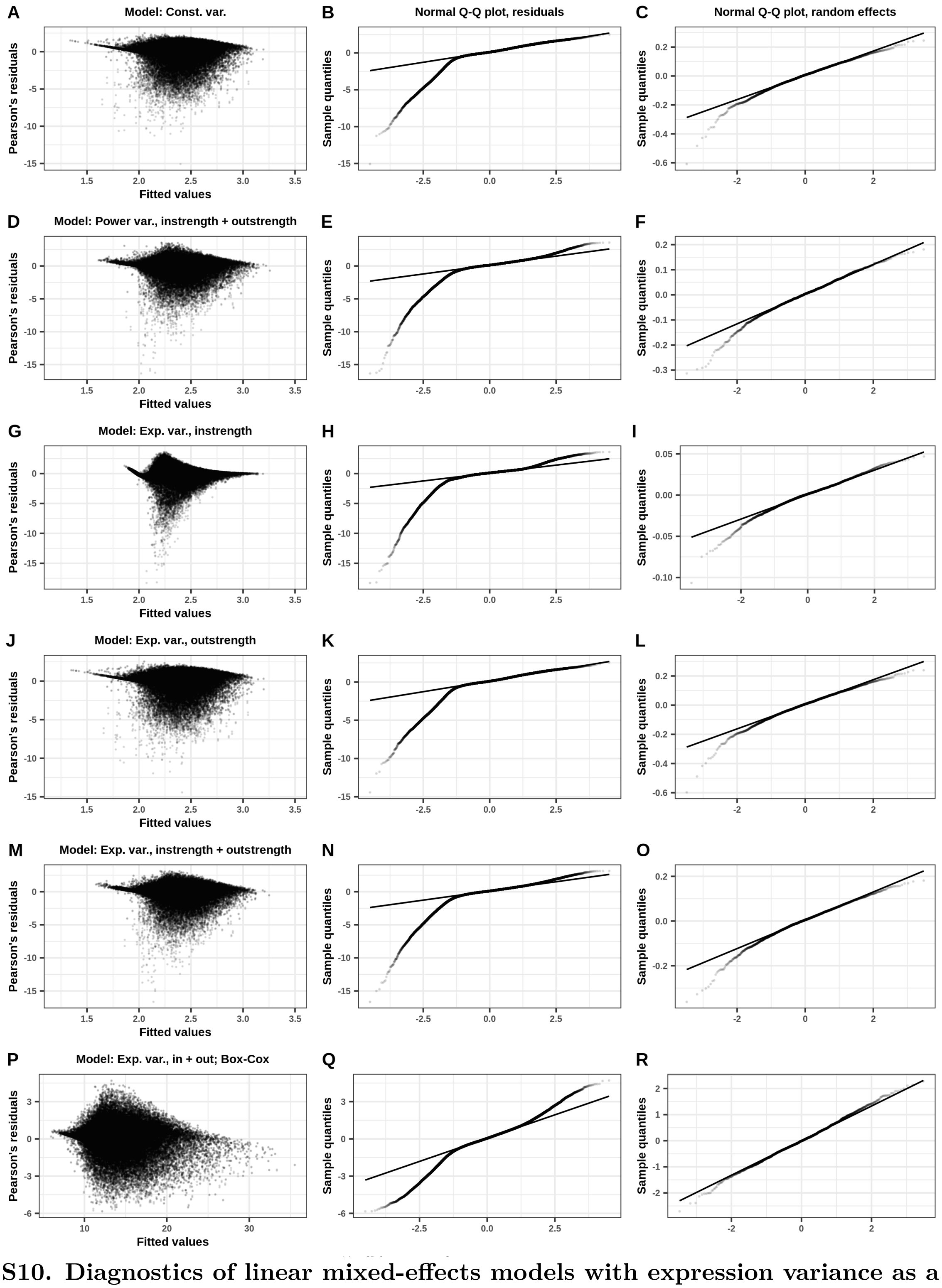

Fig S10. Diagnostics of linear mixed-effects models with expression variance as a response variable and different variance structures.

Fig S10. Continued. **A-C** Plot of Pearson's residuals vs. fitted values (A), Q-Q plot of Pearson's residuals (B), Q-Q plot of random effects (C) of a model with no variance structure. **D-F** Pearson's residuals vs. fitted values (D), Q-Q plot of standardized Pearson's residuals (E), Q-Q plot of random effects (C) of a model with a variance structure modelled as a power function of instrength and outstrength. **G-I** Pearson's residuals vs. fitted values (G), Q-Q plot of standardized Pearson's residuals (H), Q-Q plot of random effects (I) of a model with a variance structure modelled as an exponential function of instrength. **J-L** Pearson's residuals vs. fitted values (J), Q-Q plot of standardized Pearson's residuals (K), Q-Q plot of random effects (L) of a model with a variance structure modelled as an exponential function of outstrength. **M-O** Pearson's residuals vs. fitted values (M), Q-Q plot of standardized Pearson's residuals (N), Q-Q plot of random effects (O) of a model with a variance structure modelled as an exponential function of instrength and outstrength. **P-R** Pearson's residuals vs. fitted values (P), Q-Q plot of standardized Pearson's residuals (Q), Q-Q plot of random effects (R) of a model with a variance structure modelled as an exponential function of instrength and outstrength and with the explanatory variables transformed with the Box-Cox transform.

Table S2. Different variance structures do not affect the sign of the effect and significance in linear mixed-effects models with expression variance as a response variable. The results of models with different variance structures are shown in the table. The effect size differs by a small margin, but the sign and significance remain the same regardless of variance structure. The model with the variance structure as an exponential function of instrength had the lowest Akaike's Information Criterion and was chosen as the best model. Abbreviations: const. var. - constant variance; power var., in + out; variance as a power function of instrength; exp. var., in - variance as an exponential function of instrength; exp. var., out - variance as an exponential function of outstrength; exp. var., in + out - variance as an exponential function of instrength and outstrength; absInStrT_sqrt - absolute instrength, square-root transformed; absOutStrT_sqrt - absolute outstrength, square-root transformed.

Model	Predictors	Value	Std.Error	p.value	p.significant	AIC
const. var.	(Intercept)	2.12252974	0.0037040699	0.000000e+00	✔ Yes	173025.99
const. var.	absInStrT_sqrt	0.23326569	0.0014753454	0.000000e+00	✔ Yes	173025.99
const. var.	absOutStrT_sqrt	-0.07906913	0.0014530464	0.000000e+00	✔ Yes	173025.99
power var., in + out	(Intercept)	2.03654963	0.0024398194	0.000000e+00	✔ Yes	144787.06
power var., in + out	absInStrT_sqrt	0.27234172	0.0012837816	0.000000e+00	✔ Yes	144787.06
power var., in + out	absOutStrT_sqrt	-0.04355256	0.0011948879	1.415734e-289	✔ Yes	144787.06
★ exp. var., in	(Intercept)	2.01421296	0.0014226309	0.000000e+00	✔ Yes	96143.99
★ exp. var., in	absInStrT_sqrt	0.27887716	0.0011158593	0.000000e+00	✔ Yes	96143.99
★ exp. var., in	absOutStrT_sqrt	-0.02214644	0.0007327439	4.731613e-200	✔ Yes	96143.99
exp. var., out	(Intercept)	2.11543714	0.0036669065	0.000000e+00	✔ Yes	172518.76
exp. var., out	absInStrT_sqrt	0.23610694	0.0014788851	0.000000e+00	✔ Yes	172518.76
exp. var., out	absOutStrT_sqrt	-0.07599671	0.0014553914	0.000000e+00	✔ Yes	172518.76
exp. var., in + out	(Intercept)	2.05479175	0.0028207627	0.000000e+00	✔ Yes	144773.85
exp. var., in + out	absInStrT_sqrt	0.26581550	0.0013998517	0.000000e+00	✔ Yes	144773.85
exp. var., in + out	absOutStrT_sqrt	-0.04811173	0.0013049873	3.496844e-296	✔ Yes	144773.85

3.2 GLMM: Relative change of expression variance

To investigate whether local network centrality measures affect the evolution of expression variance, we fitted a linear mixed-effects model with the same formula as Eq. 1. with the normalized change of expression variance as the outcome variable. When fitting a model with the assumption of constant variance, the Pearson's residuals were heteroskedastic (Fig S11A). We fitted models with different variance structures and based on Akaike's Information Criterion chose the model with the exponential function of the node abstrength as the variance structure. Pearson's residuals of the chosen and all other fitted models are shown in Fig S11B-E. Changing the variance structure did not change the significance or the effect of the fixed variables (Table S3). The variance inflation factor (VIF), a measure of collinearity of explanatory variables, was 1.06. A VIF value lower than 3 indicates that the statistical significance of the inferred effects is reliable in spite of collinearity.

Table S3. Different variance structures do not affect the sign of the effect and significance in linear mixed-effects models with relative change of expression variance after selection as a response variable. The results of models with different variance structures are shown in the table. The effect size differs by a small margin, but the sign and significance remain the same regardless of variance structure. The model with the variance structure as an exponential function of instrength had the lowest Akaike's Information Criterion and was chosen as the best model. Abbreviations: const. var. - constant variance; power var., in + out; variance as a power function of instrength; exp. var., in - variance as an exponential function of instrength; exp. var., out - variance as an exponential function of outstrength; exp. var., in + out - variance as an exponential function of instrength and outstrength; absInStrT_sqrt - absolute instrength, square-root transformed; absOutStrT_sqrt - absolute outstrength, square-root transformed.

Model	Predictors	Value	Std.Error	DF	t.value	p.value	AIC	p.significant
const. var.	(Intercept)	0.337916277	0.0013430352	72373	251.60641	0.000000e+00	-144342.1	✔ Yes
const. var.	absInStrT_sqrt	-0.008901309	0.0004334489	72373	-20.53600	1.898873e-93	-144342.1	✔ Yes
const. var.	absOutStrT_sqrt	-0.039686466	0.0004269809	72373	-92.94669	0.000000e+00	-144342.1	✔ Yes
power var., in + out	(Intercept)	0.353325888	0.0011743341	72373	300.87339	0.000000e+00	-149555.2	✔ Yes
power var., in + out	absInStrT_sqrt	-0.013152971	0.0004173796	72373	-31.51321	1.686444e-216	-149555.2	✔ Yes
power var., in + out	absOutStrT_sqrt	-0.047848893	0.0003958800	72373	-120.86718	0.000000e+00	-149555.2	✔ Yes
★ exp. var., in	(Intercept)	0.339920854	0.0011538478	72373	294.59766	0.000000e+00	-153714.6	✔ Yes
★ exp. var., in	absInStrT_sqrt	-0.002698555	0.0004280085	72373	-6.30491	2.900180e-10	-153714.6	✔ Yes
★ exp. var., in	absOutStrT_sqrt	-0.046068532	0.0003902311	72373	-118.05450	0.000000e+00	-153714.6	✔ Yes
exp. var., out	(Intercept)	0.338768771	0.0013394715	72373	252.91227	0.000000e+00	-144376.3	✔ Yes
exp. var., out	absInStrT_sqrt	-0.009392597	0.0004342054	72373	-21.63169	1.926129e-103	-144376.3	✔ Yes
exp. var., out	absOutStrT_sqrt	-0.039910911	0.0004274997	72373	-93.35893	0.000000e+00	-144376.3	✔ Yes
exp. var., in + out	(Intercept)	0.348060641	0.0012043388	72373	289.00558	0.000000e+00	-150295.3	✔ Yes
exp. var., in + out	absInStrT_sqrt	-0.011395088	0.0004347183	72373	-26.21258	9.703240e-151	-150295.3	✔ Yes
exp. var., in + out	absOutStrT_sqrt	-0.046273751	0.0004127882	72373	-112.10047	0.000000e+00	-150295.3	✔ Yes

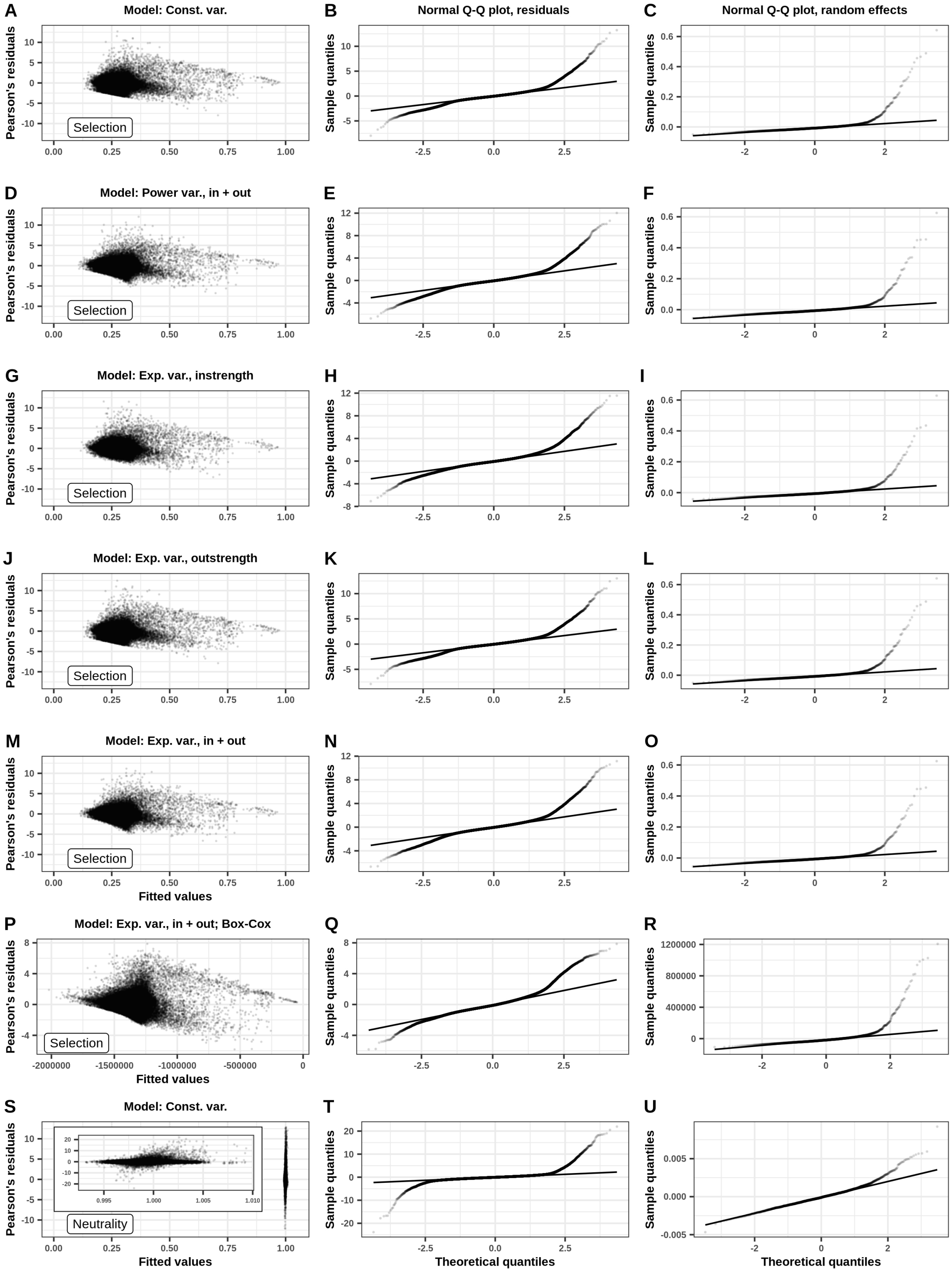

Fig S11. Diagnostics of linear mixed-effects models with relative change of expression variance after selection as a response variable and different variance structures.

Fig S11. Continued. **A-C** Plot of Pearson's residuals vs. fitted values (A), Q-Q plot of Pearson's residuals (B), Q-Q plot of random effects (C) of a model with no variance structure. **D-F** Pearson's residuals vs. fitted values (D), Q-Q plot of standardized Pearson's residuals (E), Q-Q plot of random effects (C) of a model with a variance structure modelled as a power function of instrength and outstrength. **G-I** Pearson's residuals vs. fitted values (G), Q-Q plot of standardized Pearson's residuals (H), Q-Q plot of random effects (I) of a model with a variance structure modelled as an exponential function of instrength. **J-L** Pearson's residuals vs. fitted values (J), Q-Q plot of standardized Pearson's residuals (K), Q-Q plot of random effects (L) of a model with a variance structure modelled as an exponential function of outstrength. **M-O** Pearson's residuals vs. fitted values (M), Q-Q plot of standardized Pearson's residuals (N), Q-Q plot of random effects (O) of a model with a variance structure modelled as an exponential function of instrength and outstrength. **P-R** Pearson's residuals vs. fitted values (P), Q-Q plot of standardized Pearson's residuals (Q), Q-Q plot of random effects (R) of a model with a variance structure modelled as an exponential function of instrength and outstrength and with the explanatory variables transformed with the Box-Cox transform. **S-U** Pearson's residuals vs. fitted values (M), Q-Q plot of standardized Pearson's residuals (N), Q-Q plot of random effects (O) of a model with constant variance structure fitted on the dataset of populations evolved under neutrality.

3.3 GLMM: Selective pressure

To investigate whether local network centrality measures affect the strength of selective pressure acting on genes, we fitted a linear mixed-effects model with the same formula as Eq. 1. with the selective pressure as the outcome variable. When fitting a model with the assumption of constant variance, the Pearson's residuals were heteroskedastic (Fig S12A). We fitted models with different variance structures and based on Akaike's Information Criterion chose the model with the exponential function of the node abstrength as the variance structure. Pearson's residuals of the chosen and all other fitted models are shown in Fig S12B-E. Changing the variance structure did not change the significance or the effect of the fixed variables (Table S4). The variance inflation factor (VIF), a measure of collinearity of explanatory variables, was 1.03. A VIF value lower than 3 indicates that the statistical significance of the inferred effects is reliable in spite of collinearity.

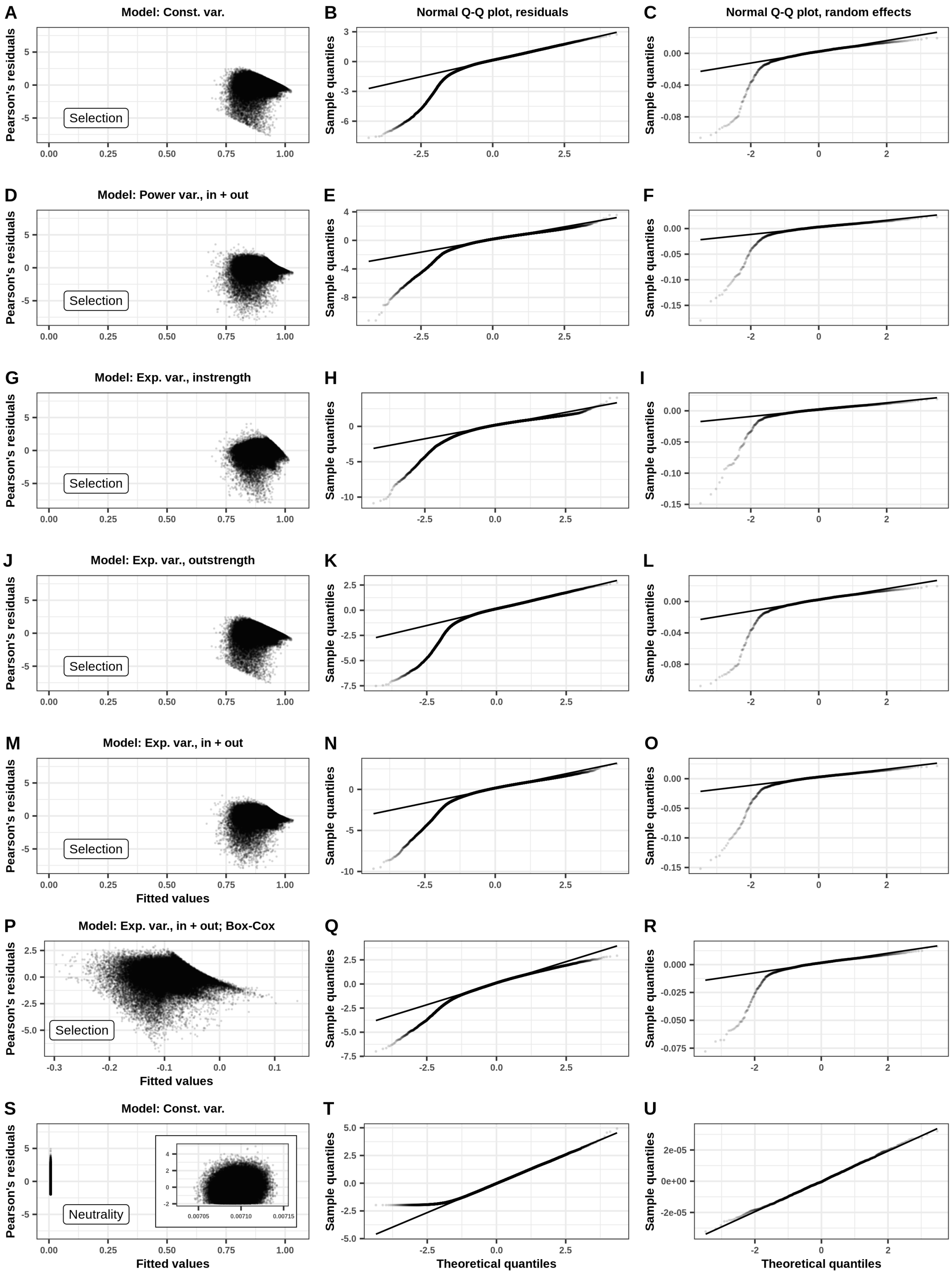

Fig S12. Diagnostics of linear mixed-effects models with selective pressure as a response variable and different variance structures.

Fig S12. Continued. **A-C** Plot of Pearson's residuals vs. fitted values (A), Q-Q plot of Pearson's residuals (B), Q-Q plot of random effects (C) of a model with no variance structure. **D-F** Pearson's residuals vs. fitted values (D), Q-Q plot of standardized Pearson's residuals (E), Q-Q plot of random effects (C) of a model with a variance structure modelled as a power function of instrength and outstrength. **G-I** Pearson's residuals vs. fitted values (G), Q-Q plot of standardized Pearson's residuals (H), Q-Q plot of random effects (I) of a model with a variance structure modelled as an exponential function of instrength. **J-L** Pearson's residuals vs. fitted values (J), Q-Q plot of standardized Pearson's residuals (K), Q-Q plot of random effects (L) of a model with a variance structure modelled as an exponential function of outstrength. **M-O** Pearson's residuals vs. fitted values (M), Q-Q plot of standardized Pearson's residuals (N), Q-Q plot of random effects (O) of a model with a variance structure modelled as an exponential function of instrength and outstrength. **P-R** Pearson's residuals vs. fitted values (P), Q-Q plot of standardized Pearson's residuals (Q), Q-Q plot of random effects (R) of a model with a variance structure modelled as an exponential function of instrength and outstrength and with the explanatory variables transformed with the Box-Cox transform. **S-U** Pearson's residuals vs. fitted values (M), Q-Q plot of standardized Pearson's residuals (N), Q-Q plot of random effects (O) of a model with constant variance structure fitted on the dataset of populations evolved under neutrality.

Table S4. Different variance structures do not affect the sign of the effect and significance in linear mixed-effects models with selective pressure as a response variable. The results of models with different variance structures are shown in the table. The effect size differs by a small margin, but the sign and significance remain the same regardless of variance structure. The model with the variance structure as an exponential function of instrength had the lowest Akaike's Information Criterion and was chosen as the best model. Abbreviations: const. var. - constant variance; power var., in + out; variance as a power function of instrength; exp. var., in - variance as an exponential function of instrength; exp. var., out - variance as an exponential function of outstrength; exp. var., in + out - variance as an exponential function of instrength and outstrength; absInStrT_sqrt - absolute instrength, square-root transformed; absOutStrT_sqrt - absolute outstrength, square-root transformed.

Model	Predictors	Value	Std.Error	DF	t.value	p.value	p.significant	AIC
const. var.	(Intercept)	0.88415564	0.0006748052	63167	1310.2382	0	✔ Yes	-189809.3
const. var.	absInStrT_sqrt	-0.04096498	0.0003106663	63167	-131.8617	0	✔ Yes	-189809.3
const. var.	absOutStrT_sqrt	0.03588198	0.0002908981	63167	123.3490	0	✔ Yes	-189809.3
power var., in + out	(Intercept)	0.87597512	0.0005514233	63167	1588.5710	0	✔ Yes	-199304.3
power var., in + out	absInStrT_sqrt	-0.03786164	0.0002801003	63167	-135.1717	0	✔ Yes	-199304.3
power var., in + out	absOutStrT_sqrt	0.03995255	0.0002456307	63167	162.6529	0	✔ Yes	-199304.3
★ exp. var., in	(Intercept)	0.88278537	0.0005377403	63167	1641.6576	0	✔ Yes	-207009.0
★ exp. var., in	absInStrT_sqrt	-0.03708753	0.0002878319	63167	-128.8513	0	✔ Yes	-207009.0
★ exp. var., in	absOutStrT_sqrt	0.03434249	0.0002328101	63167	147.5129	0	✔ Yes	-207009.0
exp. var., out	(Intercept)	0.88370457	0.0006732199	63167	1312.6537	0	✔ Yes	-189839.4
exp. var., out	absInStrT_sqrt	-0.04101737	0.0003111198	63167	-131.8379	0	✔ Yes	-189839.4
exp. var., out	absOutStrT_sqrt	0.03629719	0.0002910387	63167	124.7160	0	✔ Yes	-189839.4
exp. var., in + out	(Intercept)	0.87761056	0.0005922562	63167	1481.8090	0	✔ Yes	-199570.5
exp. var., in + out	absInStrT_sqrt	-0.03864219	0.0002999411	63167	-128.8326	0	✔ Yes	-199570.5
exp. var., in + out	absOutStrT_sqrt	0.03974518	0.0002648410	63167	150.0719	0	✔ Yes	-199570.5

3.4 GLM: Average selective pressure

To investigate whether global network centrality measures affect the average selective pressure acting on genes in networks, we fitted a linear model with the average selective pressure per network as the outcome variable, and the first two principal components as explanatory variables, after performing a principal component analysis on 12 graph-level centrality metrics. Diagnostical plots are shown in Fig S13.

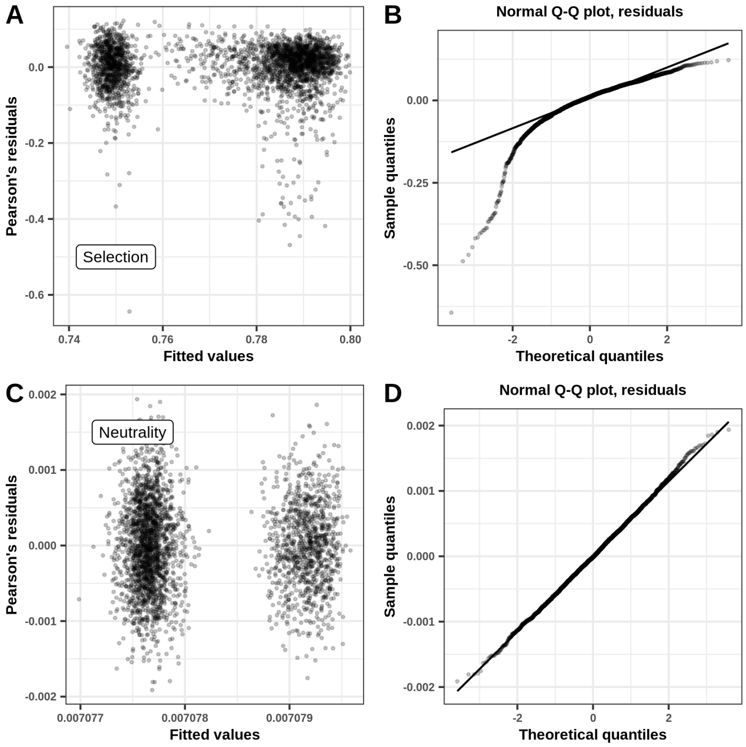

Fig S13. Diagnostics of linear model with average selective pressure per network as a response variable. A, B - Pearson's residuals vs. fitted values (A) and Q-Q plot of standardized Pearson's residuals (B) in the model fitted on selected populations. **C, D** - Pearson's residuals vs. fitted values (C) and Q-Q plot of standardized Pearson's residuals (D) in the model fitted on neutral populations.

References

1. James G, Witten D, Hastie T, Tibshirani R. An Introduction to Statistical Learning: with Applications in R. Springer Texts in Statistics. Springer US;. Available from: `https://link.springer.com/10.1007/978-1-0716-1418-1`.
