# Supplementary material for: Being noisy in a crowd: Differential selective pressure on gene expression noise in model gene regulatory networks
Source: PLoS Comput Biol. 2023 Apr 20;19(4):e1010982. doi: 10.1371/journal.pcbi.1010982 (PMC10118199; doi:10.1371/journal.pcbi.1010982)
Supplement: S4 Text — (PDF) [file pcbi.1010982.s004.pdf]

4 Robustness of results in different topology structures

The analysis of the effects of local network metrics on the evolution of expression noise was performed on a dataset of 2,000 random (Erdős–Rényi) network topologies. To check whether our results hold for other network topology types, we performed the same analysis of two additional datasets: 1,000 scale-free (Barabási–Albert) networks, and 1,000 small-world (Watts–Strogatz model) networks. The results of all generalized linear mixed-effects models and mutual information tests are consistent and summarized in Table S5.

Table S5. The effects and significance of local network centrality metrics are consistent across different topological structures. The effect size differs by a small margin, but the sign and significance remain the same across different topological structures. Dataset consists of 113,274 genes from 3,000 network topologies.

Response	Topology	Expl. var.	Beta	p-value (GLMM)[1]	MI	p-value (MI)[2]
Expression variance	ER	Instrength	0.28	$< 2.2 \times 10^{-16}$ ***	0.67	10^{-4} ***
		Outstrength	-0.02	$< 2.2 \times 10^{-16}$ ***	0.05	10^{-4} ***
	BA	Instrength	0.21	$< 2.2 \times 10^{-16}$ ***	0.34	10^{-4} ***
		Outstrength	-0.06	$< 2.2 \times 10^{-16}$ ***	0.11	10^{-4} ***
	WS	Instrength	0.25	$< 2.2 \times 10^{-16}$ ***	0.43	10^{-4} ***
		Outstrength	-0.05	$< 2.2 \times 10^{-16}$ ***	0.05	10^{-4} ***
Rel. change of expr. variance	ER	Instrength	-0.003	2.9×10^{-10} ***	0.09	10^{-4} ***
		Outstrength	-0.046	$< 2.2 \times 10^{-16}$ ***	0.14	10^{-4} ***
	BA	Instrength	-0.041	$< 2.2 \times 10^{-16}$ ***	0.19	10^{-4} ***
		Outstrength	-0.027	$< 2.2 \times 10^{-16}$ ***	0.26	10^{-4} ***
	WS	Instrength	0.004	$< 2.6 \times 10^{-7}$ ***	0.09	10^{-4} ***
		Outstrength	-0.039	$< 2.2 \times 10^{-16}$ ***	0.08	10^{-4} ***
Probability of responding to selection	ER	Instrength	-1.87	$< 2.2 \times 10^{-16}$ ***	—	—
		Outstrength	-0.08	$< 2.2 \times 10^{-16}$ ***	—	—
	BA	Instrength	-2.01	$< 2.2 \times 10^{-16}$ ***	—	—
		Outstrength	-0.4	$< 2.2 \times 10^{-16}$ ***	—	—
	WS	Instrength	-1.88	$< 2.2 \times 10^{-16}$ ***	—	—
		Outstrength	-0.16	3.9×10^{-11} ***	—	—
Gene-specific selective pressure	ER	Instrength	-0.04	$< 2.2 \times 10^{-16}$ ***	0.19	10^{-4} ***
		Outstrength	0.03	$< 2.2 \times 10^{-16}$ ***	0.31	10^{-4} ***
	BA	Instrength	-0.02	$< 2.2 \times 10^{-16}$ ***	0.14	10^{-4} ***
		Outstrength	0.02	$< 2.2 \times 10^{-16}$ ***	0.54	10^{-4} ***
	WS	Instrength	-0.04	$< 2.2 \times 10^{-16}$ ***	0.17	10^{-4} ***
		Outstrength	0.03	$< 2.2 \times 10^{-16}$ ***	0.2	10^{-4} ***

[1] Coefficients and their significance were computed using linear mixed-effects model (see Methods).
[2] Mutual information p-values were computed using a Monte Carlo permutation test with 10,000 permutations. Asterisks indicate statistical significance: n.s. - p-value > 0.05; * - p-value ≤ 0.05; ** - p-value ≤ 0.01; *** - p-value ≤ 0.001; **** - p-value ≤ 0.0001.
