# Supplementary material for: Being noisy in a crowd: Differential selective pressure on gene expression noise in model gene regulatory networks
Source: PLoS Comput Biol. 2023 Apr 20;19(4):e1010982. doi: 10.1371/journal.pcbi.1010982 (PMC10118199; doi:10.1371/journal.pcbi.1010982)
Supplement: S5 Text — (PDF) [file pcbi.1010982.s005.pdf]

5 Robustness of results to unequal fitness contribution of genes

In most simulations performed in this study we assumed for simplicity an equal fitness contribution of all genes, which is not biologically realistic. To check whether our results are robust with different parametrization of fitness contribution, we performed the same analysis of an additional dataset: 500 of the same (Erdős–Rényi) networks used in the main results, but with the values of fitness contribution of all genes $\{\rho_i\}_{1 \leq i \leq n}$ drawn from an uniform distribution $\mathcal{U}(0, 2)$. The results of all generalized linear mixed-effects models and mutual information tests are consistent and summarized in Table S6.

Table S6. The effects and significance of local network centrality metrics are consistent between assumptions of equal and unequal fitness contributions of genes. The effect size differs by a small margin, but the sign and significance remain between the two parametrizations of fitness contribution.

Response	FC[2]	Expl. var.	Beta	p-value (GLMM)[1]	MI	p-value (MI)[2]
Expression variance	equal	Instrength	0.28	$< 2.2 \times 10^{-16}$ ***	0.67	10^{-4} ***
		Outstrength	-0.02	$< 2.2 \times 10^{-16}$ ***	0.05	10^{-4} ***
	unequal	Instrength	0.26	$< 2.2 \times 10^{-16}$ ***	0.67	10^{-4} ***
		Outstrength	-0.02	$< 2.2 \times 10^{-16}$ ***	0.05	10^{-4} ***
Rel. change of expr. variance	equal	Instrength	-0.003	2.9×10^{-10} ***	0.09	10^{-4} ***
		Outstrength	-0.046	$< 2.2 \times 10^{-16}$ ***	0.14	10^{-4} ***
	unequal	Instrength	-0.006	$< 2.2 \times 10^{-16}$ ***	0.02	10^{-4} ***
		Outstrength	-0.018	$< 2.2 \times 10^{-16}$ ***	0.03	10^{-4} ***
Probability of responding to selection	equal	Instrength	-1.87	$< 2.2 \times 10^{-16}$ ***	—	—
		Outstrength	-0.08	$< 6.67 \times 10^{-7}$ ***	—	—
	unequal	Instrength	-1.96	$< 2.2 \times 10^{-16}$ ***	—	—
		Outstrength	-0.13	$< 2.2 \times 10^{-16}$ ***	—	—
Gene-specific selective pressure	equal	Instrength	-0.04	$< 2.2 \times 10^{-16}$ ***	0.19	10^{-4} ***
		Outstrength	0.03	$< 2.2 \times 10^{-16}$ ***	0.31	10^{-4} ***
	unequal	Instrength	-0.06	$< 2.2 \times 10^{-16}$ ***	0.07	10^{-4} ***
		Outstrength	0.05	$< 2.2 \times 10^{-16}$ ***	0.06	10^{-4} ***

[1] Coefficients and their significance were computed using linear mixed-effects model (see Methods).
[2] Mutual information p-values were computed using a Monte Carlo permutation test with 10,000 permutations. Asterisks indicate statistical significance: n.s. - p-value > 0.05; * - p-value ≤ 0.05; ** - p-value ≤ 0.01; *** - p-value ≤ 0.001; **** - p-value ≤ 0.0001. [2] Fitness contribution.

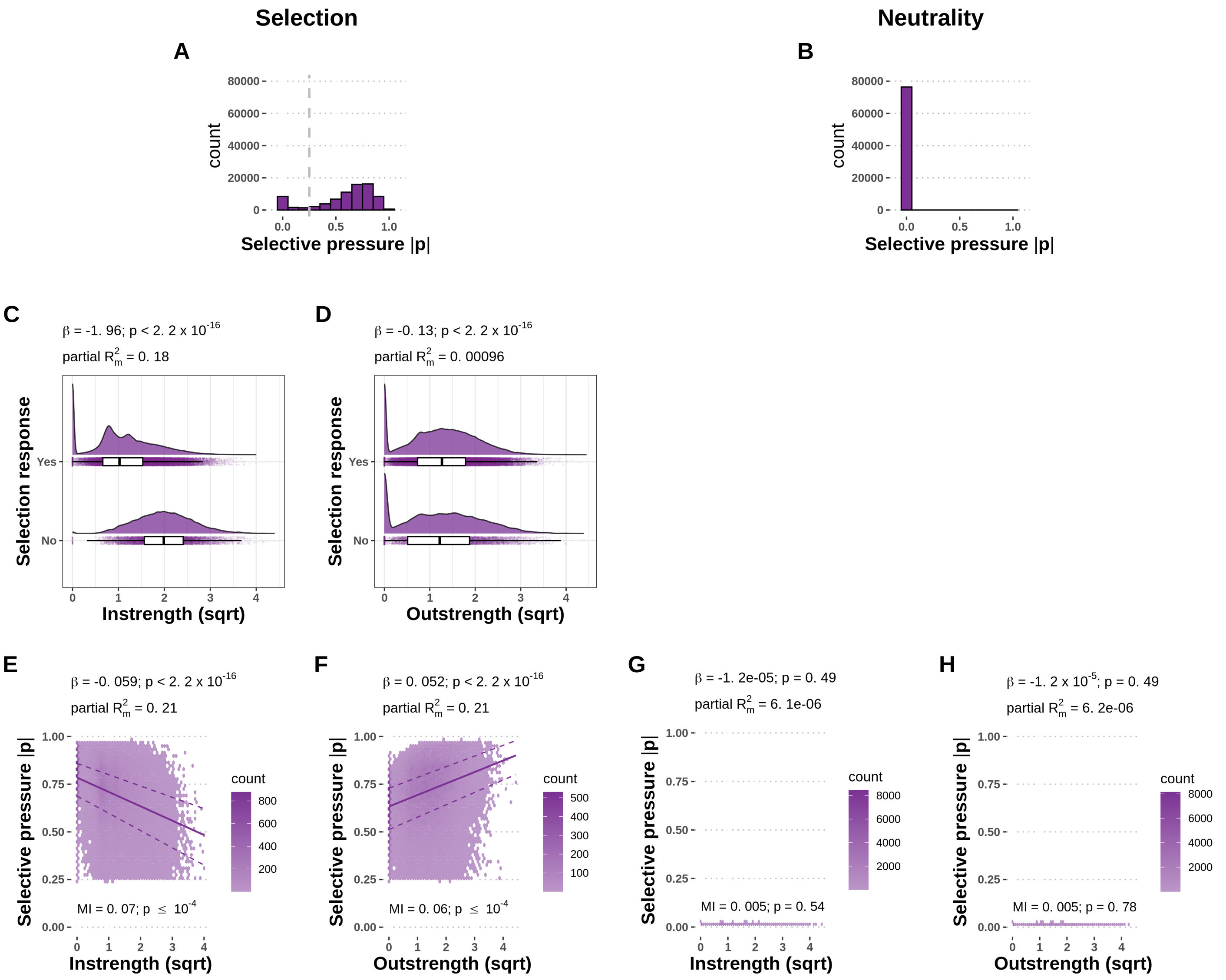

Fig S14. Differential selective pressure is acting on genes based on their centrality, regardless of the fitness contribution parametrization. A, B - Distributions of the measured selective pressure in selected (A) and neutral (B) populations. Genes with a selective pressure above 0.25 were categorized as responsive to selection. **C, D** - High instrength genes are less likely to respond to selection. Absolute instrength (C) has a strong significant negative effect on the probability of selection response. Absolute outstrength (D) has a weak significant negative effect on the probability of selection response. **E, F** - In the subset of genes that responded to selection, high instrength (E) decreases the selective pressure, while high outstrength (F) increases the selective pressure acting on individual genes. The lines indicate the 25% (lower dashed line), 50% (solid line), and 75% (upper dashed line) fitted quantiles. **G, H** - Absolute instrength (G) and outstrength (H) have no significant effect on the selective pressure in the non-selected populations. The dataset consists of 74,443 genes from 2,000 populations with unique 40-gene random network topology samples, which were independently evolved 10 times under selection and 10 times under neutrality. The selective pressure on each gene is calculated as the average normalized reduction of the intrinsic noise parameter during the evolutionary simulation and summarized as the mean over all replicates in each scenario. Coefficients, p-values and partial marginal R^2 measures are estimated using logistic regression and linear mixed-effects models with selection responsiveness or selective pressure as the response variable, instrength and outstrength as fixed effect explanatory variables, and the network topology sample as the random effect explanatory variable. Mutual information (MI) p-values were computed using 10,000 permutations.
