# Supplementary material for: Being noisy in a crowd: Differential selective pressure on gene expression noise in model gene regulatory networks
Source: PLoS Comput Biol. 2023 Apr 20;19(4):e1010982. doi: 10.1371/journal.pcbi.1010982 (PMC10118199; doi:10.1371/journal.pcbi.1010982)
Supplement: S6 Text — (PDF) [file pcbi.1010982.s006.pdf]

6 Filtered datasets

We performed the analyses on two additional datasets to get a clearer picture of the effects of instrength and outstrength on the expression noise metrics. The first filtered dataset is a dataset in which genes that are both regulators and regulated were removed, *i.e.* it consists exclusively of pure regulators (genes that regulate others and are not being regulated) and purely regulated genes (genes that are being regulated and do not regulate other genes). The second filtered dataset is a dataset that consists exclusively of genes that are both regulators and regulated, *i.e.* in this dataset pure regulators and purely regulated genes have been removed. The effects and significance of the two local centrality metrics are consistent in analyses of expression variance, relative change of expression variance and gene-specific selective pressure (Table S7). In the unfiltered and second filtered dataset we found a significant small negative effect of outstrength on the probability of responding to selection. However, the negative effect is lost when we analysed only the genes that are either regulators or regulated genes (Filtered 1 dataset), and we observed a significant strong positive effect of outstrength on the probability of responding to selection. We concluded that instrength in genes that are both regulators and regulated influences the effect of outstrength on the probability of responding to selection and that there are complex interactions between the two centrality metrics. However, when there are no genes that have both instrength and outstrength the effects are clear and instrength has a strongly negative effect, while outstrength has a strongly positive effect on the probability of a gene to respond to selection.

Table S7. Filtered and unfiltered datasets. Filtered 1 dataset is dataset in which genes that are both regulators and regulated were removed, *i.e.* it consists exclusively of pure regulators (genes that regulate others and are not being regulated) and purely regulated genes (genes that are being regulated and do not regulate other genes). Filtered 1 dataset consists of 43,214 genes from 2,000 random network topologes. Unfiltered dataset consists of 148,886 genes from 2,000 random network topologes. Filtered 2 dataset consists exclusively of genes that are both regulators and regulated, *i.e.* in this dataset pure regulators and purely regulated genes have been removed. Filtered 2 dataset consists of 105,672 genes from 2,000 random network topologes.

Response	Dataset	Expl. var.	Beta	p-value (GLMM)[1]	MI	p-value (MI)[2]
Expression variance	Filtered 1	Instrength	0.29	$< 2.2 \times 10^{-16}$ ***	0.8	10^{-4} ***
		Outstrength	7.8×10^{-4}	$< 2.6 \times 10^{-9}$ ***	0.61	10^{-4} ***
	Unfiltered	Instrength	0.28	$< 2.2 \times 10^{-16}$ ***	0.67	10^{-4} ***
		Outstrength	-0.022	$< 2.2 \times 10^{-16}$ ***	0.05	10^{-4} ***
	Filtered 2	Instrength	0.24	$< 2.2 \times 10^{-16}$ ***	0.43	10^{-4} ***
		Outstrength	-0.08	$< 2.2 \times 10^{-16}$ ***	0.02	10^{-4} ***
Rel. change of expr. variance	Filtered 1	Instrength	-0.033	$< 2.2 \times 10^{-16}$ ***	0.29	10^{-4} ***
		Outstrength	-0.073	$< 2.2 \times 10^{-16}$ ***	0.34	10^{-4} ***
	Unfiltered	Instrength	-0.003	2.9×10^{-10} ***	0.09	10^{-4} ***
		Outstrength	-0.046	$< 2.2 \times 10^{-16}$ ***	0.14	10^{-4} ***
	Filtered 2	Instrength	-0.017	$< 2.2 \times 10^{-16}$ ***	0.07	10^{-4} ***
		Outstrength	-0.035	$< 2.2 \times 10^{-16}$ ***	0.08	10^{-4} ***
Probability of responding to selection	Filtered 1	Instrength	-1.94	$< 2.2 \times 10^{-16}$ ***	—	—
		Outstrength	1.55	$< 9.79 \times 10^{-11}$ ***	—	—
	Unfiltered	Instrength	-1.87	$< 2.2 \times 10^{-16}$ ***	—	—
		Outstrength	-0.08	$< 6.67 \times 10^{-7}$ ***	—	—
	Filtered 2	Instrength	-1.79	$< 2.2 \times 10^{-16}$ ***	—	—
		Outstrength	-0.25	$< 2.2 \times 10^{-16}$ ***	—	—
Gene-specific selective pressure	Filtered 1	Instrength	-0.05	$< 2.2 \times 10^{-16}$ ***	0.63	10^{-4} ***
		Outstrength	0.03	$< 2.2 \times 10^{-16}$ ***	0.72	10^{-4} ***
	Unfiltered	Instrength	-0.04	$< 2.2 \times 10^{-16}$ ***	0.1	10^{-4} ***
		Outstrength	0.03	$< 2.2 \times 10^{-16}$ ***	0.05	10^{-4} ***
	Filtered 2	Instrength	-0.04	$< 2.2 \times 10^{-16}$ ***	0.1	10^{-4} ***
		Outstrength	0.03	$< 2.2 \times 10^{-16}$ ***	0.17	10^{-4} ***

[1] Coefficients and their significance were computed using linear mixed-effects model (see Methods).
[2] Mutual information p-values were computed using a Monte Carlo permutation test with 10,000 permutations. Asterisks indicate statistical significance: n.s. - p-value > 0.05; * - p-value ≤ 0.05; ** - p-value ≤ 0.01; *** - p-value ≤ 0.001; **** - p-value ≤ 0.0001.
