# Supplementary material for: Being noisy in a crowd: Differential selective pressure on gene expression noise in model gene regulatory networks
Source: PLoS Comput Biol. 2023 Apr 20;19(4):e1010982. doi: 10.1371/journal.pcbi.1010982 (PMC10118199; doi:10.1371/journal.pcbi.1010982)
Supplement: Attachment — Submitted filename: m1_rebuttal_letter.pdf [file pcbi.1010982.s007.pdf]

Response to review comments for submission PCOMPBIOL-D-22-01212

Title: Being noisy in a crowd: differential selective pressure on gene expression noise in model gene regulatory networks

Nataša Puzović[1*], Tanvi Madaan [1], Julien Y. Dutheil[1,2]
[1] Molecular Systems Evolution Research Group, Max Planck Institute for Evolutionary Biology,

Plön, Schleswig-Holstein, Germany
[2] Institut des sciences de l'évolution, Montpellier, Languedoc-Roussillon, France
Corresponding Author: puzovic@evolbio.mpg.de

We thank the editor and the reviewers for their valuable feedback. We recalled all reviewers points below (in italic) and numbered our answers accordingly. We edited the manuscript to clarify the points raised by the reviewers, emphasizing our initial hypothesis and streamlining the discussion. We also included a new set of simulations with unequal gene contributions to fitness.

Reviewer 1:

R1.1:

> *"In this manuscript, Puzovic, Madaan, and Dutheil present a stochastic gene-regulatory network to analyze the evolution of gene expression noise in single cells. They build on an existing model, extend it to generate expression noise and benchmark the model through a number of scenarios. The methodology is described rigorously (I miss this in a good fraction of papers), and results should be reproducible and also something to build upon for future work."*

A1.1: We thank the reviewer for their positive assessment of our methodology.
R1.2:

> *"Having said that, I do not think that the current version of the manuscript meets the scope and requirements for publication in PLoS Comp Biol. My main concern is that the current results are more of a description/demonstration/benchmarking of the model, and lacks links to biological systems, or new insight overall. I do appreciate, however, the extent of the simulation data generated, yet for me (a bit outside of the "network biology" sub-discipline) this is not enough to convince of the usefulness of such models. I encourage the Authors to think of ways to use their current work for learning new biology (rather than putting a short discussion on putative implications in the Discussion section), and resubmit a head-to-toe improved and updated version."*

A1.2: We regret that our extensive benchmark shadowed the original question for which we designed this model. In this work, we specifically aim at understanding which selective forces can induce genes to evolve distinct amount of expression noise, our working hypothesis being that selection for low noise at the network level would lead to distinct selective pressures at the

gene level, depending on the underlying network structure. We designed a new model in order to test this hypothesis. This model has two essential features: interactions between genes in the form of a regulatory network, and evolvable intrinsic expression noise. Because of the novelty of the model, we first assess its properties, particularly its capacity to reproduce known biological features such as noise propagation. In the revised version of our manuscript, we further recall our working hypothesis (presented in the introduction, lines 105-107) in the results and discussion sections (see lines 356-357 and 594-597). The discussion part, which has been streamlined (see reviewer 3's comment) now states:

> In this work, we aimed at understanding how natural selection shaped the distribution of expression noise levels between genes in the genome. We hypothesized that selection for low noise at the network level translates into differential selective pressures at the gene level. To test this hypothesis, we developed a new gene regulatory network evolution model that incorporates stochastic gene expression, where the gene expression mean and variance are both heritable and, therefore, potentially subject to natural selection. We simulated the evolution of gene-specific expression noise in populations of model gene regulatory networks under selective and non-selective conditions. In agreement with our hypothesis, we observed that individual genes respond differently to the global selective pressure and that this response depends on the local and global network properties.

We believe this new version to be more focused, and the biological questions to be more straightforwardly stated.

Reviewer 2:

R2.1

> *"In this manuscript, the authors have studied the evolution of gene-specific expression noise in gene networks. The motivation for this work is to explore how in phenotypes, central genes have shown to have lesser expression noise compared to peripheral genes. The authors have formulated forward-in-time simulation framework to study the evolution of stochastic gene expression in phenotypes. This is done by imposing stabilization selection and evolving networks through steps of mutation, selection, and recombination. The authors showed that the peripheral genes may have higher expression noise due to increased selective pressure on central genes leading to noise amplification to peripheral genes.*
>
> *Furthermore, the authors have studied the impact of local and global network features on the expression noise. The authors report higher centrality metrics leads to stronger selective pressure to reduce expression noise.*
>
> *Overall, this manuscript is interesting, and the methods used are clearly explained. The topic studied in this manuscript is of importance in understanding the evolution of gene networks.*
>
> *The authors have provided thoughtful supplementary information.*

I had a few comments while reading the manuscript.

Is selective pressure defined in eq 3 ("the strength of imposed selective pressure is set to be identical for all constituent genes equals to 1 for all genes" lines 193:194) different from selective pressure defined in line 253 (average change of noise in every second generation)? Lines 193:194 suggests selective pressure is "imposed" to be equivalent to 1 and the same for all."

A2.1 The "selective" pressure referred to in lines 193:194 and line 253 are, indeed, different. To avoid ambiguity, we have re-termed the first "selective pressure" as "fitness contribution". The fitness contribution (previously "selective pressure" in lines 193:194) defines the contribution of the expression level of each gene to the fitness of the individual, *i.e.* it is a scaling factor of the decrease of fitness as a function of the distance of the expression level from the optimal expression level. For example, for the same deviation of the measured expression level from the optimal expression level $|s_i^{opt} - s_i| = 10$, a gene whose contribution to fitness is $\rho_i = 0.05$ will reduce the fitness by $\Delta f = 0.99$, while a gene whose contribution to fitness is $\rho_i = 1.95$ will reduce the fitness by $\Delta f = 0.12$ (*Eq. 3*). The fitness contribution is equal and set to 1 for all genes in all networks in our dataset (but see answer **A3.1** to Reviewer 3's comment). Consequently, deviations of the expression level from the optimal expression level are equally penalized for all genes. On the other hand, what we termed "selective pressure" in line 253 is defined as the average change of noise genotypes throughout the evolutionary simulation relative to the starting level of intrinsic noise. In the populations evolved under neutrality, the selective pressure is, expectedly, close to 0 (Fig. 5B). In the populations evolved under selection, the selective pressure has a bimodal distribution with the higher peak close to 1 (Fig. 5A). Even though the fitness contribution of the expression level of each individual gene is equal, there are differences in the selective pressure when evolved under stabilizing selection on all genes. To summarize, the contribution of fitness is a parameter defined at the beginning of the evolutionary simulation, and the selective pressure is measure of response to selection inferred from the simulation results.

For clarity, we have added a sentence to further explain the "contribution of fitness" parameter in the main text (lines 206-209).

R2.2:

"Line 214: Does under the network establishment step, steady state expression level of genes converge to the optimum level? If they do not, then what may be the reason for not converging to steady state? It may be beneficial to point out this."

A2.2: The steady state expression level of genes of the fittest network at the end of the network establishment step converges to the optimum expression level range $\frac{s_{max}}{2} \pm \frac{s_{max}}{4}$ in 68% (54333/80000) of the genes (Fig. S3). We noticed that about 7% (5557/80000) of genes are either uniformly switched off (their expression level is 0) or maximally expressed (their expression level is s_{max}) in the entire population even with noisy gene expression, indicating they are frozen components of the system and, consequently, these genes were filtered out from the dataset used in the analysis (Methods section, lines 271-272). Reasons why not all genes converge to the imposed optimal expression level during the network establishment include: 1) potential constraints in the network configuration, *e.g.* a gene with no upstream regulators will not be able to change its mean expression

level, as the basal expression level is immutable during the evolutionary simulation; 2) the optimization/fitting algorithm is stuck in a local sub-optimum where the expression level of some genes in the network did not converge to the imposed optimal expression level. We note, however, that even though not all genes reach the imposed optimal expression level of $s = \frac{s_{max}}{2}$, most of them have intermediate expression levels at the end of the network establishment process, and, more importantly, the optimal expression levels for the noise evolution simulations is set as the steady state expression levels of the fittest network at the end of the network establishment process. In other words, the optimal expression levels in the noise evolution simulations reflect the steady state of the established network, not the initial optimal expression levels from the network establishment process.

For clarity, we have added further explanations of the steady state expression levels of the networks at the end of the network establishment process and potential reasons for convergence failure (lines 267-271), and a subsection to the SI (Section 1.3).

R2.3:

> *"Would it be beneficial to define Instrength and Outstrength as the summation of real regulatory strength values $\sum_j W_{ij}$, as compared to the summation of absolute regulatory strength? If considered summation of real regulatory strength, then it will be beneficial to differentiate between if a gene is overall activated or repressed. I acknowledge this might be out of the scope of this manuscript."*

A2.3: We thank the reviewer for this suggestion. While the regulatory strengths are indeed signed values, only the absolute value of the strength impacts gene expression noise in the context of network propagation: noise is propagated from upstream to downstream genes regardless of the whether the regulator is a repressor or an activator. As only the absolute regulatory strength is relevant for noise propagation, we considered the sum of the absolute regulatory strength values when computing instrength and outstrength. We further note that using the sum of the real regulatory strength values as a measure of network centrality would not allow to distinguish genes with no interactions from genes where negative and positive interactions would be symetric, leading to a sum close to 0. For example, a gene that equally activates one gene and represses another gene would have an identical sum of real regulatory strengths as a gene which does not regulate any other gene. The expression noise of the first hypothetical gene is propagated downstream while the second is not, leading to two very distinct predictions for the same centrality value.

Reviewer 3:

> *"The authors extend a previous gene network model and perform corresponding evolutionary simulations to test the hypothesis that the expression noise of highly connected genes in gene regulatory networks is under stronger selective pressure than the expression noise of less connected genes. This work builds on previous studies that have shown that the gene networks modulate expression noise and evolve under selective pressure. The manuscript is well written and the figures are acceptable. Though the main finding is novel, interesting, and timely, I have several major concerns, largely related to the assumptions made in the modelling, that must be addressed before I can accept this manuscript for publication in PLoS Computational Biology.*

Major Concerns

Several of the model assumptions are not biologically realistic. For instance, imposing the same selective pressure for all genes ignores the benefits/disadvantages that the expression of one gene might have relative to another gene. I also suspect that the relationship between expression noise levels and mean fitness of the network is not biologically realistic; specific levels of noise for one gene with similar "outstrength" and "instrength" as another gene, may lead to different effects to the mean fitness between the two genes. Finally, as gene regulatory networks evolve, it does not seem that the immutable condition made for the regulatory interactions is valid. The following additional scenarios should be simulated and analyzed: **R3.1:** *1) different selective pressures for different genes in a given nework,* **R3.2:** *2) network architectures with different levels of mean fitness from the evolved populations (see comment below),* **R3.3:** *3) in-silico evolution simulations for different populations sizes, a key parameter in evolution (not just N = 1000 (where computationally feasible); and* **R3.4:** *4) gene networks to evolving to change their regulatory interactions during the noise evolution step in the evolutionary simulation process."*

A3.1: We agree with the reviewer that modeling genes with distinct contributions to fitness would be more biologically realistic. This requires, however, to specify an *a priori* distribution of these effects. To test whether the assumption of homogeneous contributions has an effect on our conclusions, we added another set of simulations with 2000 network samples and with the fitness contribution for each gene drawn from a uniform distribution $\mathcal{U}(0, 2)$ instead of being set to 1 for all genes. The average fitness contribution across genes in the added simulation is 1 and is identical to the average fitness contribution of the previous simulations, but this parametrization allows the genes within each network to have different contributions to fitness. We ran the same analysis pipeline as in the main results. We find that the distribution of the inferred selective pressure in the populations with fitness contributions drawn from the uniform distribution (Fig. S14A) has a higher variance than the inferred selective pressure in the populations with equal fitness contribution in all genes (Fig. 5A), which is expected. Our conclusions from the previous simulations are unaffected – high instrength genes have lower probability of responding to selection, high instrength reduces the selective pressure, while high outstrength increases selective pressure (Fig. S14). Notably, the effect size of the network centrality metrics on the selective pressure is higher in the populations with unequal fitness contributions than in populations with equal fitness contributions.

We have added the results of the added simulation to the supplementary material as Section 5 titled "Robustness of results to unequal fitness contribution of genes" and refer to it in the main text (lines 210-214).

A3.2: We respond in the comment below, where additional information is provided.

A3.3: We have performed simulations with six different population sizes, from $N = 50$ to $N = 2000$ (SI Section 1.4, Fig. S4). We observe that the average selective pressure is lower in smaller population sizes, and increases with the population size. This is predicted by population genetics theory, which states that mutations with a selection coefficient below $1/N$ are effectively neutral. Therefore, increasing population size *de facto* increases the proportion of mutations visible to selection. We observe that this effect plateaus after $N = 1000$, after which increasing the population

size only marginally increases further the selective pressure. Therefore, we have chosen $N = 1000$ as a trade-off between selective pressure and computational efficiency.

A3.4: We agree with the reviewer that allowing the network to change during the evolutionary course is a more biologically realistic perspective. Such a more complex scenario is indeed possible to implement within our general framework. However, we note that allowing the regulatory interactions to mutate would allow the mean expression levels to evolve, as well as the local centrality metrics of individual genes and the global network architecture. This would mean that instead of having one evolvable trait, the expression noise levels, with fixed mean expression levels and fixed local and global network topology, four traits would be evolving simultaneously. This would make the underlying analysis significantly more complex, notably to assess whether one trait drives – or responds to – selection on another. In the scope of this study, we are specifically interested in expression noise, which we believe to be the less well understood and studied trait of the four, asking how the other factors impact its evolution and, therefore, fixing them during the *in silico* evolution experiment. We emphasize this original question in the revised manuscript (see also Reviewer's 1 comment and answer). The coevolution of expression noise and expression mean in different selective scenarios is something we are currently exploring in a follow-up project.

R3.2 (continued):

> *"Related to the comment above, the authors found that gene expression noise is reduced under a stabilizing selection regime, which is expected since the noise evolution simulations were performed for the gene network configurations with the highest fitness under stabilizing selection. Accordingly, since these gene networks achieved close to optimal fitness, any deviations from this due to gene expression noise is selected against and gene expression noise reduced. I would like to see simulations of scenarios where the evolution for gene networks that do not start close to the optimal fitness/scenarios where gene expression noise is beneficial (as is mentioned in the Introduction) and for different types of selection (i.e., disruptive selection and negative selection), where the outcomes are less intuitive. For the latter, at a minimum add a discussion on what you anticipate would occur if disruptive and negative selection were incorporated into your model."*

A3.2: Expression noise is indeed expected to be generally reduced under stabilizing selection. It has been theoretically predicted and experimentally demonstrated in previous studies (*e.g.* Duveau et al. (2018)). A striking experimental observation, however, is that different genes have distinct noise levels, and the noise level correlates with gene features, so expression noise levels are not randomly distributed in the genome. In this work, we argue that it is necessary to consider the system at which stabilizing selection occurs. We hypothesize and demonstrate that selection at the gene network level translates into differential selective pressures at the gene level, via the regulatory network topology. We further emphasized this hypothesis in the revised version of the manuscript.

We agree with the reviewer that network topology most likely impacts noise evolution in other types of selection scenarios, such as directional, disruptive or fluctuating, and that our general framework can be useful in this context. For the scope of this paper, however, we chose to restrict our study to stabilizing selection, which we believe to be the most common scenario, to

understand the evolution of differential expression noise at the gene level. Studying the coevolution of expression noise and mean in other selective scenarios is an exciting perspective we are currently working on.

R3.5:

> *"Several important works on gene network architecture and the evolution of expression noise have not been mentioned/cited in this manuscript. Specifically, at a minimum, the following theoretical/computational and experimental studies from Mads Kaern, Gabor Balazsi, and Csaba Pal groups should be incorporated into the Introduction and Discussion sections of this manuscript: Charlebois, Phys. Rev. E, 2014 2015; Bodi, PLoS Biol., 2017; Camellato, Eng. Biol., 2019; and Farquhar, Nat. Commun., 2019."*

A3.5: We are thankful for these references and have added the mentioned studies to the manuscript.

R3.6:

> *"Minor Concerns*
>
> *Introduction: Define stabilizing selection for unfamiliar readers."*

A3.6: We added a definition of stabilizing selection to the manuscript (lines 200-203). The corresponding sentence now reads:

> Stabilizing selection, the selection scenario in which individuals with extreme phenotypic values have a lower fitness, was imposed on all constituent genes by setting the value of optimal expression level as the mean equilibrium expression level of each gene.

R3.7:

> *"Methods & Results: I suggest removing the deterministic model from this manuscript, as it is confusing and seems out of place for a study focusing on gene expression noise. If it is essential to leave in the deterministic model, emphasize why this is so in the manuscript."*

A3.7: The deterministic model is a particular case of our general framework, with the noise parameter set to 0. It is used for benchmarking, but also in the network establishment process.

For clarification, we have added a specification of when each model is used to the manuscript (lines 170-173):

> The deterministic realization has been used to benchmark the model and to set up the mean expression levels for the starting populations, and the stochastic realization has been used in the main bulk of the simulations, in which intrinsic noise is evolved.

R3.8:

> *Results & Discussion: I suggest combining these sections to reduce repetiveness and to reduce the length of the manuscript. Alternatively, move all discussion of the results to the Discussion section.*

A3.8: We have made major adjustments to the Results and Discussion section to avoid repetition and reduce the length of the manuscript. Several paragraphs and one section has been removed from the Discussion, which now focuses on the general perspective and implications of our results. More specific comments on our results have been moved to the Results section.

R3.9:

> *"Line 127: Based on the way the regulatory matrix is defined I assume that the genes in your model can autoregulate (though I did not see autoregulation in the figures)?"*

A3.9: The model allows for autoregulation, as defined in the main diagonal in the regulatory matrix. In this study, however, we chose not to include autoregulation (*i.e.* the main diagonal in all network samples is always zero) because negative and positive autoregulation is known to decrease and increase expression noise, respectively, and would therefore be a confounding factor when analysing the effect of network centrality on selective pressure acting on gene-specific expression noise.

In the revised version, we explicitly state that autoregulation is not present and the reason for its omission in the manuscript (lines 186-187).

R3.10:

> *"Lines 144-146: A justification and/or citation(s) should be provided for the assumption that there is no cooperative or competitive binding of transcription factors to transcription factor binding sites. Any limitations that result from making this assumption should also be discussed."*

A3.10: Cooperative or competitive binding of transcription factors to transcription factor binding sites is common in nature and adds a degree of non-linearity to the promoter activity of the regulated gene with respect to the concentration of the transcription factor. In our model, this relationship is assumed to be linear. In fine-grained modelling of gene regulation, the promoter activity of a gene regulated with cooperative or competitive transcription factors is usually modelled with a Hill function. The parameter determining the steepness of the function and reflecting the transcription factor cooperation or competition is the Hill coefficient. A higher Hill coefficient indicates a more step-like function. Generally, the Hill coefficient is in the range of $n_{Hill} \in [1, 4]$, which corresponds to moderately steep functions (Alon, 2019), which are well approximated by a linear function. We note that our model can be extended to include a Hill function for every gene, which would add more parameters to the model (for which *a priori* values would be needed) and increase the computation time. The limitation of assuming no cooperative or competitive binding among transcription factors is that the non-linear response to transcription factor concentrations in a certain range at the extremes (extremely low or extremely high concentration) could be a mechanism of suppressing noise in this range, but this phenomenon has not been well studied.

For clarity, we have added a justification and limitation of the assumption of no cooperative or competitive binding among transcription factors (lines 143-148):

> The cumulative effect of all transcription factors in the expression level of each gene is for simplicity considered to be additive, *i.e.* we assume there is no cooperative or competitive binding of transcription factors to transcription factor binding sites. This

> assumption removes the small degree of non-linearity in the response of the regulated gene to transcription factor concentrations, which is present in real transcription factor regulation dynamics.

R3.11:

> *"Lines 151-153: Is the using the normal distribution for gene expression justified? What about for bimodal gene expression that can occur for genes regulated by positive feedback gene networks (e.g., Nevozhay, PLoS Comput. Biol., 2012)? Same question for drawing the noise genotype mutations (Lines 201-202) and regulatory strength mutations (Lines 223-224) from a normal distribution."*

A3.11: The normal distribution is used to compute the value of the gene expression level in one time point during the phenotype realization process. We implemented a Gaussian noise as a simple form of isotropic noise, with a single parameter. We note that this assumption does not necessarily imply that the distribution of gene expression levels in the clonal population is normal or even unimodal. The expression levels of each individual is the product of the trajectory of the expression levels during the realization process, during which expression levels can exhibit phenotypic switching between stable states. Consequently, there can be a bimodal expression level distribution of a certain gene in the clonal population, even though the expression levels in each time step are drawn from a normal distribution.

In the revised version, we provide more details on the implication of the normality assumption and comment on non-normal expression level distributions in the clonal population (lines 153-164). We further add a justification for the noise genotype and regulatory strength mutation distributions (lines 222-226):

> There is no experimental evidence for the shape of the distribution of the expression noise and regulatory strength mutations. We chose a normal distribution because: 1) it defines equally frequent beneficial and deleterious mutations and 2) most mutations have a small effect, which reflects the characteristic of many studied distributions of fitness effects in model organisms.

R3.12:

> *"Lines 198-200: Gene expression in the absence of a selective agent can be costly (again see Nevozhay, PLoS Comput. Biol., 2012), yet it is assumed that there there is an equal constant value for the fitness of all phenotypes. This should be mentioned in the Discussion section of the manuscript."*

A3.13: Gene expression costs have, indeed, not been taken into consideration in this study. We have added a note on the costs of expression affecting gene expression distributions (lines 624-625).

R3.14:

> *"Lines 227 – 228: Discuss the limitations of removing regulatory configurations that resulted in oscillating gene expression levels."*

A3.14: We added the following sentences to the corresponding Methods section (lines 252-260):

> Network regulatory configurations in which the expression level of all genes would not converge to a fixed point and would oscillate were discarded, as in previous studies (Laarits et al., 2016). Oscillating gene expression level patterns create population-level heterogeneity generated by the system oscillations and not by stochastic gene expression. Since we are studying the evolution of gene-specific expression noise, expression noise generated by oscillations would be a confounding factor in our analysis. We note, however, that oscillatory networks can be frequent in simulations (Pinho et al., 2012) and biological systems (Zhang et al., 2014), and the role of expression noise in their behavior is an interesting perspective for follow-up studies.

R3.15:

> *"Lines 560-561: "GLMMs, however, make several assumptions that might be violated by the data in some cases. To further validate our conclusions, we computed the mutual information (MI) between variables." Explicitly state which assumptions are being referred to and why mutual information avoids these assumptions/provides more robustness."*

A3.15: We are referring here to the Gauss-Markov assumptions of linear models: normality, homoskedasticity and independence of the error terms. We have clarified these assumptions in the revised version of the manuscript and clarified how using MI (a non parametric measure) as an additional metric avoids the problems of potential assumption violation (lines 346-350):

> Generalized linear mixed-effects models make several assumptions that might be violated by the data in some cases. Namely, they assume a normal distribution and homoskedasticity of Pearson's residuals, and a normal distribution of random effects. To further validate our conclusions, we computed the mutual information (MI) between variables, which does not have any prior assumptions.

References

U. Alon. *An Introduction to Systems Biology*. Chapman and Hall/CRC, Boca Raton, Fla, 2nd edition edition, Aug. 2019. ISBN 978-1-4398-3717-7.

F. Duveau, A. Hodgins-Davis, B. P. Metzger, B. Yang, S. Tryban, E. A. Walker, T. Lybrook, and P. J. Wittkopp. Fitness effects of altering gene expression noise in Saccharomyces cerevisiae. *eLife*, 7:e37272, Aug. 2018. ISSN 2050-084X. doi: 10.7554/eLife.37272. URL `https://elifesciences.org/articles/37272`.

T. Laarits, P. Bordalo, and B. Lemos. Genes under weaker stabilizing selection increase network evolvability and rapid regulatory adaptation to an environmental shift. *Journal of Evolutionary Biology*, 29(8):1602–1616, Aug. 2016. ISSN 1010061X. doi: 10.1111/jeb.12897. URL `http://doi.wiley.com/10.1111/jeb.12897`.

R. Pinho, E. Borenstein, and M. W. Feldman. Most Networks in Wagner's Model Are Cycling. *PLoS ONE*, 7(4):e34285, Apr. 2012. ISSN 1932-6203. doi: 10.1371/journal.pone.0034285. URL `https://dx.plos.org/10.1371/journal.pone.0034285`.

R. Zhang, N. F. Lahens, H. I. Ballance, M. E. Hughes, and J. B. Hogenesch. A circadian gene expression atlas in mammals: Implications for biology and medicine. *Proceedings of the National Academy of Sciences*, 111(45):16219–16224, Nov. 2014. doi: 10.1073/pnas.1408886111. URL https://www.pnas.org/doi/10.1073/pnas.1408886111. Publisher: Proceedings of the National Academy of Sciences.
